# Single and combined nanotoxicity of titanium dioxide and cadmium: implications for skeletal and behavioral development in zebrafish

**DOI:** 10.1007/s11356-026-38005-2

**Published:** 2026-07-15

**Authors:** Carolina Castro, Joseph Mamboungou, Gabriel Qualhato, Felipe Cirqueira, Thiago Lopes Rocha, Lucélia Gonçalves Vieira

**Affiliations:** 1 Institute of Biological Sciences, Federal University of Goiás, Avenida Esperança S/N, Campus Samambaia, Goiânia, Goiás 74690-900 Brazil; 2 Institute of Tropical Pathology and Public Health, Federal University of Goiás, Goiânia, Goiás Brazil; 3 School of Veterinary Medicine and Animal Husbandry, Federal University of Goiás, Goiânia, Goiás Brazil

**Keywords:** Fish, Nanomaterials, Chondrotoxicity, Osteotoxicity, Embryotoxicity, Trojan Horse effect

## Abstract

Titanium dioxide nanoparticles (TiO_2_ NPs) are emerging contaminants of global concern. However, knowledge about their interactive effects with metals on developing aquatic vertebrates is still scarce. Thus, the current study investigated cartilage and bone toxicity and behavioral changes in *Danio rerio* (zebrafish) larvae after exposure to single and combined TiO_2_ NPs (0.1 µg L^−1^, 1.0 µg L^−1^, and 10 µg L^−1^) and Cd (10 µg L^−1^) for 168 h. The results showed that environmentally relevant concentrations of single TiO_2_ NPs did not cause skeletal changes in zebrafish larvae. In contrast, single Cd and combined with TiO_2_ NPs induced significant skeletal changes, such as malformation of chondrocranial structures and inhibition of ossification. Despite that, single TiO_2_ NPs or combined with Cd did not induce changes in the locomotor behavior of zebrafish larvae. This study demonstrated the chondrotoxicity and osteotoxicity of Cd, single, and combined with TiO_2_ NPs, on zebrafish, highlighting the potential ecotoxicological impact of co-exposure to inorganic nanoparticles and metals on aquatic organisms, especially on the development of the skeletal system.

**Graphical abstract:**

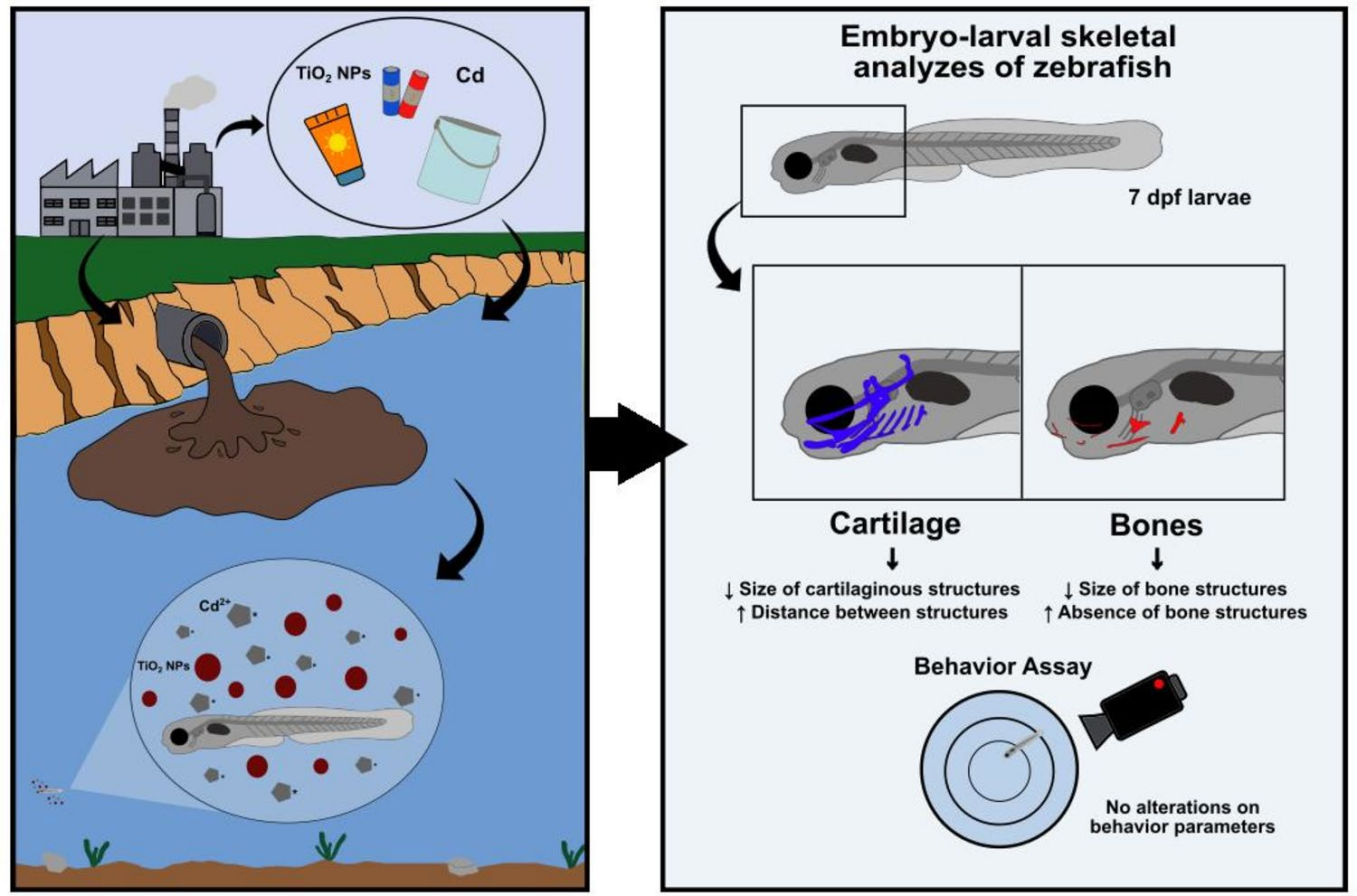

## Introduction

Titanium dioxide nanoparticles (TiO_2_ NPs) are increasingly used in the food industry, medicine, plastics production, cosmetics, and paint pigments (Waghmode et al. 2019). With their widespread use, TiO_2_ NPs are commonly present in water bodies, domestic sewage and industrial effluents (Chang et al. 2017; Slomberg et al. 2020). Under experimental conditions, TiO_2_ NPs were very harmful to flora and fauna, such as the aquatic plants *Spirodela polyrrhiza* (Movafeghi et al. 2018), the microcrustacean *Daphnia magna* (Zhu et al. 2010), fish *Hoplias intermedius* (Disner et al. 2017) and *Acipenser schrenckii* (Zhou et al. 2024), and amphibian *Duttaphrynus melanostictus* (Ruvinda & Pathiratne, 2021). Therefore, improper disposal in the aquatic ecosystem can lead to interaction with organisms and other contaminants, including cadmium (Cd) (Abd-Elhakim et al. 2021).

In the aquatic environment, TiO_2_ NPs can become carriers of traditional and emergent contaminants, facilitating and enhancing the entry of environmental chemicals into the cells of organisms, which can lead to changed toxicity. This interaction can be called the Trojan Horse effect (Naasz et al. 2018) and was reported for lead (Pb) (Fan et al. 2017), arsenic (As) (Qian et al. 2020), and Cd (Huang et al. 2021). It has also been reported in zebrafish with other nanoparticles (NPs), such as polystyrene nanoplastics (PS NPs) carrying methylmercury (MeHg) (Oger et al. 2025), carbon-based nanoparticles (CB NPs) carrying benzo(α)pyrene (B(α)P) (Binelli et al. 2017), and nanoplastics and zinc oxide nanoparticles (ZnO NPs) facilitate Cd accumulation (Chen et al. 2025). It has already been observed that TiO_2_ NPs can alter the bioavailability and bioaccumulation of Cd in aquatic organisms (Della Torre et al. 2015). The Cd bioaccumulation in zebrafish can be accentuated in the presence of TiO_2_ NPs (Hu et al. 2011). TiO_2_ NPs can bioaccumulate in carp (*Cyprinus carpio*) and facilitate Cd entry into the organism (Zhang et al. 2007). Furthermore, single and combined exposure of TiO_2_ NPs and Cd for 96 h induced genotoxicity in the marine mussel *Mytilus galloprovincialis*, reducing the genomic template stability and causing DNA damage (Rocco et al. 2015).

Cd is a non-essential metal and, as a result of technological advances, is increasingly present in the environment, as it is used in the production of dyes, plastics, corrosion-resistant alloys, metal coatings, fertilizers, and batteries (Genchi et al. 2020). Cd is easily transported in aqueous media, such as industrial wastewater, sewage, and agricultural runoff, and can enter the environment in large quantities (Wright & Welbourn 1994). When present in the environment, Cd can penetrate the body of animals and cause immunotoxicity (Zheng et al. 2016), reproductive toxicity (Tian et al. 2020), histopathological damages and oxidative stress (Lacave et al. 2020), and affect the physiology of the skeletal system (Tarasco et al. 2019) and behavior (Han et al. 2019).

The skeletal system plays an important role in the support and locomotion of animals, and its complete formation, which involves tightly regulated physiological and metabolic processes, is essential (Bird & Mabee 2003). Its development is based on complex patterns of gene expression organized temporally (Dover 2000; Wagner & Karsenty 2001). Damage to this process, whether physical or chemical, can lead to morphofunctional changes that affect animal behavior. For example, 20-day-old zebrafish (*Danio rerio*) larvae exposed to Cd showed significant skeletal deformities, such as reduction in operculum growth and deformation of the vertebrae, arches, fins, and rays (Tarasco et al. 2019). This has already been reported for juvenile fish *Gambusia affinis*, also exposed to Cd at different temperatures (24 °C and 32 °C), causing marked vertebral column deformity (Sassi et al. 2010). In *Bufo gargarizans* tadpoles, chronic exposure to Cd and Pb, single and combined, caused a reduction in the formation of endochondral bone. The Cd/Pb combined had a more severe impact on the larvae (Chen et al. 2022a). In another study, which investigated the effects of parental exposure for 4 days to the CdSe/ZnS quantum dots (QDs) combined on the skeletal development of the F1 generation of *Gobiocypris rarus*, there was an impact on the development of the cartilage of the F1 larvae, affecting the chondrocytes and altering the length and angle of the cartilaginous structures of the chondrocranium (Chen et al. 2022b). Due to the physicochemical similarities between Cd^2+^ and calcium ion (Ca^2+^), Cd can compete with this ion and cause alterations in calcium signalling pathways, leading to toxic effects in organisms, especially when it comes to ossification, since calcium plays an important role in the bone mineralization process (Choong et al. 2014).

Given the limited knowledge regarding the combined effects of TiO₂ NPs and Cd on zebrafish development, particularly concerning skeletal formation and neurobehavioral responses, this study assessed cartilage and bone integrity, as well as behavioral changes in zebrafish larvae following 168 h of exposure to TiO₂ NPs and Cd, single and combined. Considering that both contaminants can interfere with developmental and physiological pathways, we hypothesized that co-exposure induce skeletal deformities and behavioral disturbances. This study may contribute to a broader understanding of the ecological risks associated with the simultaneous presence of nanoparticles and metals in aquatic environments.

## Methodology

### TiO_2_ NPs and cadmium

TiO_2_ NPs (individual diameter ≤ 100 nm, anatase and rutile) (CAS: 13463–67-7) and cadmium chloride (CdCl_2_) (CAS: 7440–43-9) were obtained from Sigma-Aldrich. The exact anatase/rutile ratio of the commercial TiO₂ NPs (Sigma-Aldrich, product no. 634662) is unknown, as the manufacturer did not provide this information and no independent X-ray diffraction (XRD) analysis was conducted to discover this ratio. This represents an important limitation of this study, as the crystalline phase composition is a key determinant of TiO₂ NP toxicity and cannot be inferred from the biological data alone. The morphological characteristics of the TiO_2_ NPs were previously analyzed by Transmission Electron Microscopy (TEM) (Jeol, JEM-2100, equipped with EDS, Thermo scientific) by Mamboungou et al. (2022). The hydrodynamic diameter, polydispersity index (PDI), and surface charge (zeta potential) of the TiO_2_ NPs, single, or combined with Cd in ultrapure water and reconstituted water (exposure medium) were analyzed by dynamic light scattering (DLS) and electrophoretic light scattering (ELS) in the Zetasizer (Malvern Panalytical). Higher concentrations than those used in the bioassay were used for characterization due to the limitations of the equipment for detecting these NPs; thus, the analysis was not carried out using the water used in the experiment itself. Therefore, 20 mg L^−1^ of each solution/dispersion was used. The TiO_2_ NPs stock dispersion and Cd solution (Cd^2^^+^), using cadmium chloride [CdCl_2_. 5/2H_2_O], was prepared in Milli-Q water (100 mg L^−1^) and sonicated in ultrasound QUIMIS Q335D2 for 30 min, as described by Rocha et al. (2014).

### Zebrafish (*Danio rerio*)

Adult zebrafish, AB strain, aged between 6 and 18 months, were obtained and maintained at Multi-User Center for Animal Production and Experimentation (CMPEA) of the Institute of Tropical Pathology and Public Health (IPTSP) of the Federal University of Goiás (UFG). The animals were kept in polycarbonate aquaria (Rack ZEBRAFISH - ALTAMAR^®^), with a constant water flow under a continuous water temperature controlled at 26 ± 1 °C, pH of 7.0 ± 0.5, dissolved oxygen levels above 80% and following a light:dark photoperiod of 14:10 h, according to the recommendations established by Dammski et al. (2011). The animals’ daily diet consisted of commercial feed (for tropical fish - Poytara^®^) and *Artemia* sp. *nauplii* (Avdesh et al. 2012). The maintenance of adult animals and the execution of the embryotoxicity test with zebrafish embryos and larvae (ZELT) followed the regulations of OECD Guide No. 236 (OECD 2013), with approval by the Animal Use Committee of the Federal University of Goiás (CEUA-UFG n. 069/2021).

### Toxicity tests

The exposure was carried out according to the recommendations of Lammer et al. (2009). To this end, adult male and female zebrafish were moved to breeding tanks for reproduction (Alesco^®^) in a 2:1 ratio. After breeding, the embryos were collected and transferred to a Petri dish, then washed thoroughly with reconstituted water (94.0 mg L^−1^ CaCl_2_.2H_2_O; 123.3 mg L^−1^ MgSO_4_.7H_2_O; 63.0 mg L^−1^ NaHCO_3_; 5.5 mg L^−1^ KCl) (ISO 1996). Embryos, less than 4 h after fertilization, were assessed and selected for viability using a photomicroscope (Stemi 508) according to the protocols described by Kimmel et al. (1995) and Lammer et al. (2009).

**Fig. 1:**
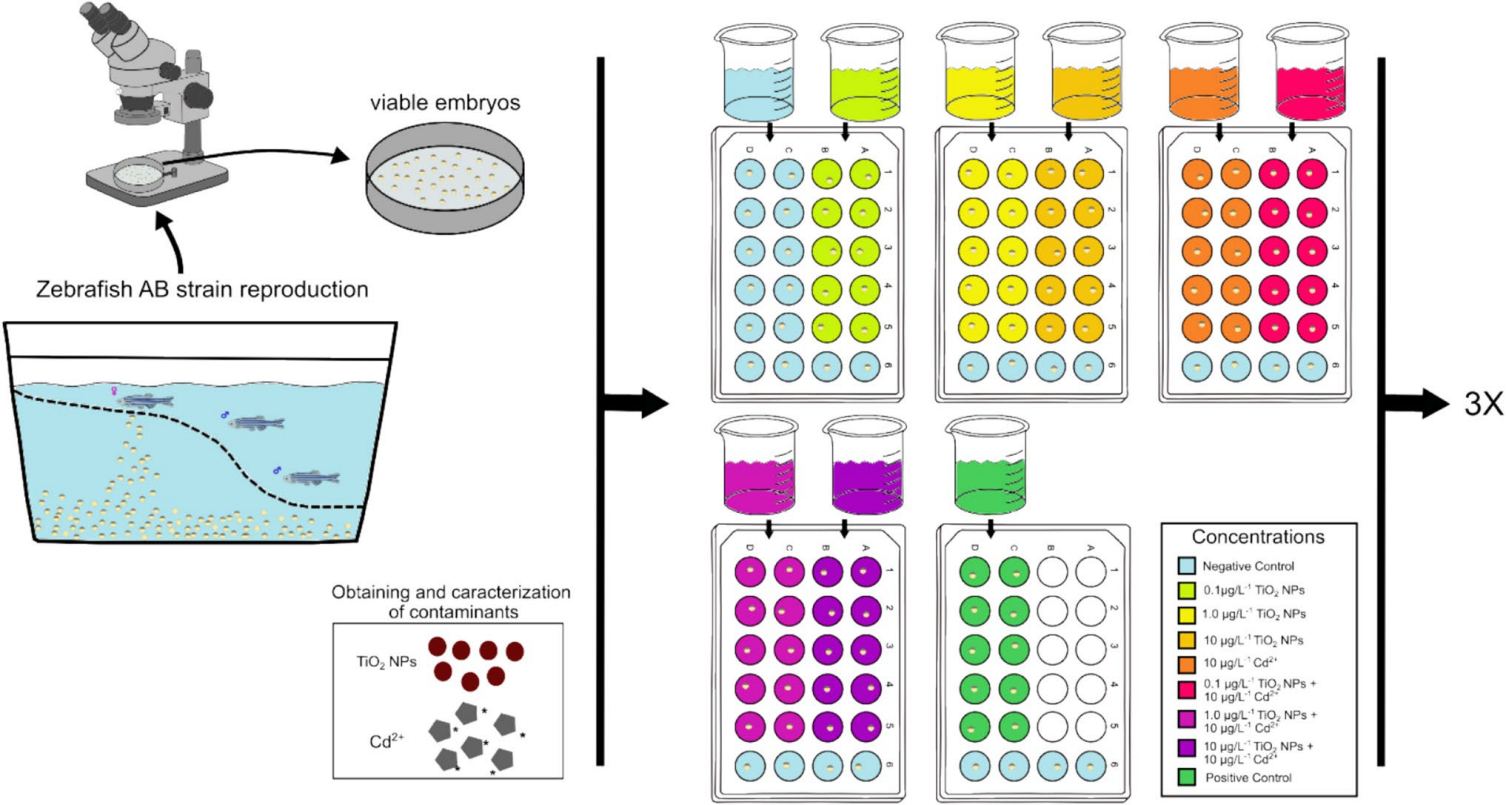
Illustrated diagram of the experimental design for exposure of zebrafish embryos and larvae to titanium dioxide nanoparticles (TiO_2_ NPs) and cadmium (Cd^2+^)

The zebrafish embryo and larva toxicity test (ZELT) protocol was performed during 168 h of exposure, in triplicate. The viable embryos were placed in 24-well microplates (Kasvi^®^), keeping 1 embryo per well containing 2 mL of exposure medium. Each microplate contained two experimental groups, with 10 embryos per group, totalling 30 embryos per experimental group. Two tests were performed, using semi-static conditions (renewal of the exposure medium every 24 h, after 30 min of sonication in a bath) for 168 h, under controlled pH and temperature conditions in a BioOxygen Demand (BOD) incubator (SOLAB SL-224/120, Brazil), the animals were not fed during the experiment. The following experimental groups were investigated: TiO_2_ NPs groups (0.1 µg L^−1^, 1.0 µg L^−1^, and 10 µg L^−1^); Cd group (10 µg L^−1^); combined groups (0.1 µg L^−1^ TiO_2_ NPs and 10 µg L^−1^ Cd; 1.0 µg L^−1^ TiO_2_ NPs and 10 µg L^−1^ Cd; 10 µg L^−1^ TiO_2_ NPs and 10 µg L^−1^ Cd); negative control group (reconstituted water) and positive control group (3,4-dichloroaniline at 3.7 mg L^−1^) (OECD 2013) (Fig. 1). All concentrations of TiO_2_ NPs were determined based on the group’s previous work (Mamboungou et al. 2022) and the data available in the literature for zebrafish (Bar-Ilan et al. 2013) and microorganisms (Dedman et al. 2021). These studies used the same concentrations, which were toxic to the organisms tested. These are predicted environmental concentrations, due the difficulty of sampling and characterising nanopolutants at low concentrations; there is a lack of environmental data. The environmentally relevant concentration for Cd was determined based on the standards established for the discharge of Brazilian effluents and reported ambient concentrations worldwide (Iqbal et al. 2013; Pekey et al. 2004; Suthar et al. 2009; Yao et al. 2014). According to National Environmental Council (CONAMA) Resolution 357/2005, the maximum permitted limit for Cd is 10 µg L^−1^. It is worth pointing out that this regulation, enacted in 2005 (Brasil 2005), aims to protect water resources and preserve the quality of the environment, ensuring that industrial activities and other sources of pollution do not cause significant damage to human health and aquatic ecosystems.

The mortality rate was analyzed daily during 168 h of exposure; embryos with advanced coagulation, failure to detach the tail, defective somite formation, absence of heartbeat, and spontaneous contractions were considered dead (ISO 1996; Lammer et al. 2009; OECD 2013). Tests were considered valid when the survival rate of the control group was > 90%.

### Evaluation of skeletal development

#### Skeletal preparation

To analyze the cartilaginous structures and the ossification process, cartilage and bone were cleared and stained according to the method by Dingerkus and Uhler (1977) with modifications. Staining was carried out separately to avoid decalcification or overlapping of ossification centers by chondrocranium structures, which at this stage of development are much more advanced in relation to ossification. After the behavioral analysis recordings, they were euthanized by hypothermia and 20 zebrafish larvae per experimental group (10 for cartilage analysis and 10 for bone analysis) were fixed in 3.7% paraformaldehyde in 0.2 M phosphate-buffered saline (PBS) at pH 7.2 for 2 h.

#### Cartilage staining

After fixation, the larvae were washed in distilled water for 3 h (three baths of one hour each) to remove paraformaldehyde residues. To stain the cartilage, 0.1% Alcian blue 8GX dye was used and dissolved in an ethanol solution and glacial acetic acid in a ratio of 8:2, respectively, for 12 h. This dye is a specific cartilage marker due to its affinity with the sulfated polysaccharides present in the cartilage matrix (Mahecha and Oliveira 1994; Scott and Dorling 1965). Afterwards, the larvae were rinsed in ethanol and gradually rehydrated in decreasing alcohol solutions (80, 70, 40, and 15%). The larvae were immersed in saturated sodium tetraborate solution for 4 h for neutralization. The soft tissues were clarified in a 0.5% KOH solution for 4 h. To minimize pigmentation of the larvae, a 3% hydrogen peroxide solution was used in a 0.5% KOH solution. After passage in increasing glycerol solutions, the larvae were kept in pure glycerol at room temperature until the analysis.

#### Staining of ossification centers

After fixation and washing in distilled water for 3 h (three baths of one hour each) to remove paraformaldehyde residues, the larvae were dehydrated in 80% ethanol for 3 h. Subsequently, the larvae were immersed in a staining solution with 0.5% alizarin for 30 min. The alizarin red S dye was used in this stage of bone staining, as it has an affinity with the calcium ions in the bone matrix (Moriguchi et al. 2003). To minimize pigmentation of the larvae, a 3% hydrogen peroxide solution was used in a 0.5% KOH solution for 30 min. After passage in increasing glycerol solutions, the larvae were kept in pure glycerol at room temperature until the analysis.

#### Skeletal structure assessment method

To assess chondro and osteotoxicity, the zebrafish larvae were analyzed under a STEMI 508 stereo microscope (Zeiss) coupled to an image capture system (ZEISS - Axiocam 105 color), using ZEN 2.6 software. The terminology of the cartilaginous structures was based on the work of Schilling et al. (1996), Kimmel et al. (2001), and Walker and Kimmel (2007), and bone structures according to Strecker et al. (2013). The method for assessing skeletal development after cleared and stained followed the parameters of Strecker et al. (2013). The measurements of the structures were made in the software Image J. The changes were categorized for statistical analysis:

- Score 0 (no changes): presented as the control group;
- Score 1 (mild to moderate alterations): all skeletal structures were present, but with incomplete development, such as the presence and degrees of chondrification and ossification centers at a delayed stage compared to the control group; presentation of shape and size of skeletal structures that correspond to an earlier stage of development.
- Score 2 (severe alterations): Absence of formation of skeletal structures; presence of structures with twists and/or undulations in the skeletal elements.

The condition index of changes in the skeletal system (IC esq) was determined for each zebrafish larva according to Figueirêdo et al. (2025). To do this, the degree of development of each skeletal structure (relative weight—RW) was classified into three categories: zero (absent), 1 (incomplete development), and 2 (complete development). Next, the IC esq will be determined according to the following formula (Figueirêdo et al. 2025):

$$ICesq=n{\sum}_{j=1}DjOj$$

where ICesq = condition index of skeletal changes; Dj = weight relative to the development of the structures; Oj = Boolean variable ranging from zero (absent) to 1 (present); *n* = total number of skeletal structures analyzed.

### Behavioral analysis

Behavioral analysis was conducted following the protocols outlined by Pinheiro-Da-Silva et al. (2020). At 144 h of exposure, a total of 15 larvae (5 larvae per replicate) from each experimental group were transferred to 24-well microplates (KASVI^®^) containing 2 mL of reconstituted water (ISO 1996) for acclimation to room temperature before recording began. To obtain the footage, an adapted recording system was used (Puluz Photo Light Box^®^) and a webcam (Logitech C922 Pro^®^) was attached to the top with a top view of the microplate. The behavior of the larvae was recorded for 1 min.

The computerized video analysis used the ZebTrack software developed by Pinheiro-Da-Silva et al. (2017) in MATLAB (R2016a; MathWorks, Natick, MA). The locomotor behavioral parameters assessed were average speed (cm/s), maximum speed (cm/s), total distance travelled (cm), and time traveled in the periphery (s). To assess exploratory behavior, visual guides were placed under the microplates consisting of two concentric circles containing an inner and outer area with dimensions of 1 cm in diameter for the inner area and 2.5 cm for the outer area, by which the time spent, distance traveled, and speed in each of the regions were measured.

### Statistical analysis

Statistical analyses were carried out using R on the Integrated Development Environment (IDE) RStudio (version 3.5.2), with parametric and non-parametric tests, assumption tests like Shapiro-Wilk and Levene test were used to assess the distribution and homogeneity of variances to verify if the data were parametric or non-parametric. ANOVA one-way and the Tukey post hoc test were carried out for parametric data, and for non-parametric data, the Kruskal-Wallis and Dunn test were the chosen methods. For mortality analysis, the log-rank test and the post hoc pairwise were done. All graphs were generated using GraphPad Prism 8 software. Results were considered significant when *p* < 0.05.

## Results and discussion

### Characteristics of TiO_2_ nanoparticles

DLS measurements revealed that the hydrodynamic diameter of TiO_2_ NPs in distilled water was 403.46 ± 11.05 nm and in reconstituted water was 781.56 ± 147.32 nm. Furthermore, ELS analysis showed that TiO_2_ NPs have a negative surface charge in distilled and reconstituted water conditions, with values of − 16.29 ± 0.47 mV and − 4.14 ± 0.16 mV, respectively, confirming that zebrafish embryos and larvae may have been exposed to aggregates/clusters of TiO_2_ NPs with a negative surface charge (Mamboungou et al. 2022). These results highlight the influence of the aqueous medium on the stability of TiO_2_ NPs, with a significant increase in particle size in reconstituted water compared to distilled water. This tendency to aggregate or agglomerate can be attributed to various factors, including electrostatic interactions and solvent characteristics (MacCuspie et al. 2011). However, the lack of an analysis of the crystallinity of the TiO_2_ NPs is a significant limitation. Such an analysis could confirm the crystalline phase of the NPs and complement the results relating to aggregation, as different crystallization phases, anatase, or rutile, may exhibit variations in size depending on the medium (Barnard & Curtiss 2005; Sukarman et al. 2024). These variations may influence the potential toxicity of these TiO₂ NPs.

TiO_2_ NPs acquire a positive surface charge in the presence of Cd, 15.03 ± 0.66 mV in distilled water and 12.24 ± 2.44 mV in reconstituted water. This alteration in zeta potential is associated with changes in the hydrodynamic diameters of the TiO_2_ NPs + Cd in both aqueous media, with values of 628.33 ± 92.42 nm for distilled water and 821.06 ± 55.82 nm for reconstituted water. In addition, the PDI values of 0.19 ± 0.09 nm for distilled water and 2.04 ± 1.38 nm for reconstituted water, when TiO_2_ NP is isolated, and 0.78 ± 0.69 nm for distilled water and 2.22 ± 0.16 nm for reconstituted water, when TiO_2_ NP is combined with Cd, confirmed the formation of TiO_2_ NP aggregates/agglomerates with different hydrodynamic diameters, single or combined with Cd, as Hartmann et al. (2012) and Xu (2018) reported.

### Mortality

Zebrafish larvae exposed for 168 h to TiO_2_ NPs and Cd, single or combined, did not show changes in mortality rate in comparison with negative control (> 10% of mortality; *p* > 0.05) and positive control (80% of mortality; *p* < 0.05). Similar results have already been observed in other studies, such as in zebrafish larvae exposed to 10, 100, and 1000 μg L^−1^ of TiO_2_ NPs for 144 h (Gu et al. 2021). On the other hand, higher levels of Cd are associated with more evident toxicity, as larvae exposed to Cd at 30.66 and 61.32 μg L^−1^ showed increased mortality (Han et al. 2019).

### Skeletal toxicity of TiO_2_ NPs and Cd

**Fig. 2:**
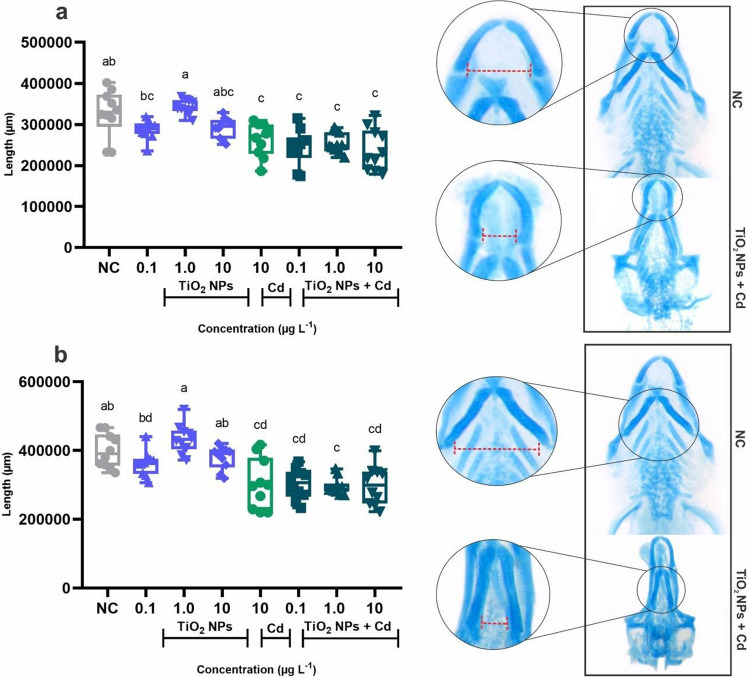
Changes in the chondrocranium of zebrafish larvae after exposure to titanium dioxide nanoparticles (TiO_2_ NPs) and cadmium (Cd), single or combined, for 168 h under semi-static conditions. **a** Distance between the branches of MK cartilage; **b** distance between the branches of the CH cartilage. Different letters represent statistically different groups (*p* < 0.05)

**Fig. 3:**
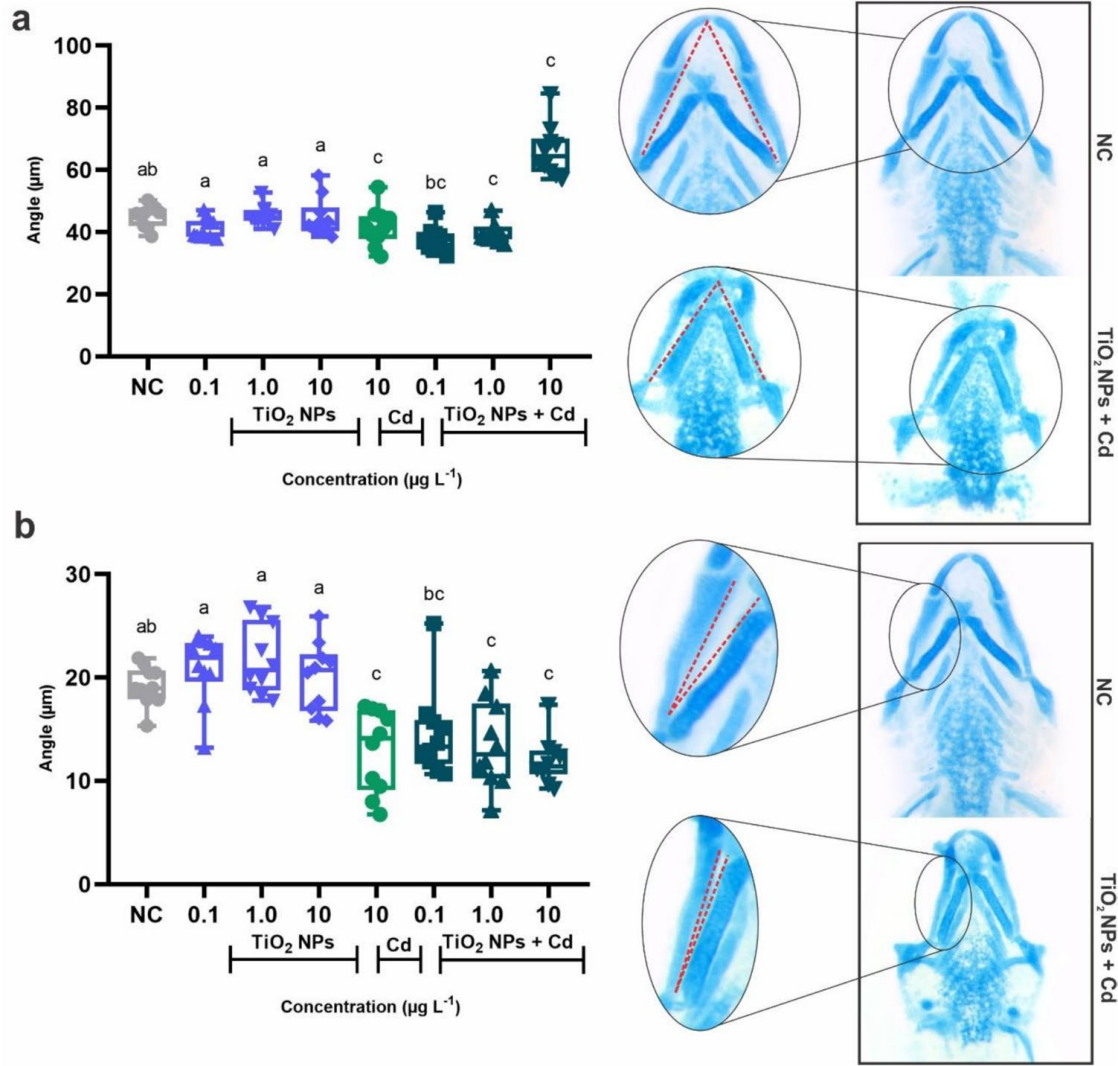
Chondrocranium of zebrafish after exposure to titanium dioxide nanoparticles (TiO_2_ NPs) and cadmium (Cd), single or combined, for 168 h under semi-static conditions. **a** Angle between MK and PQ cartilages; **b** angle between PQ and CH cartilages. Different letters represent statistically different groups (*p* < 0.05)

**Fig. 4:**
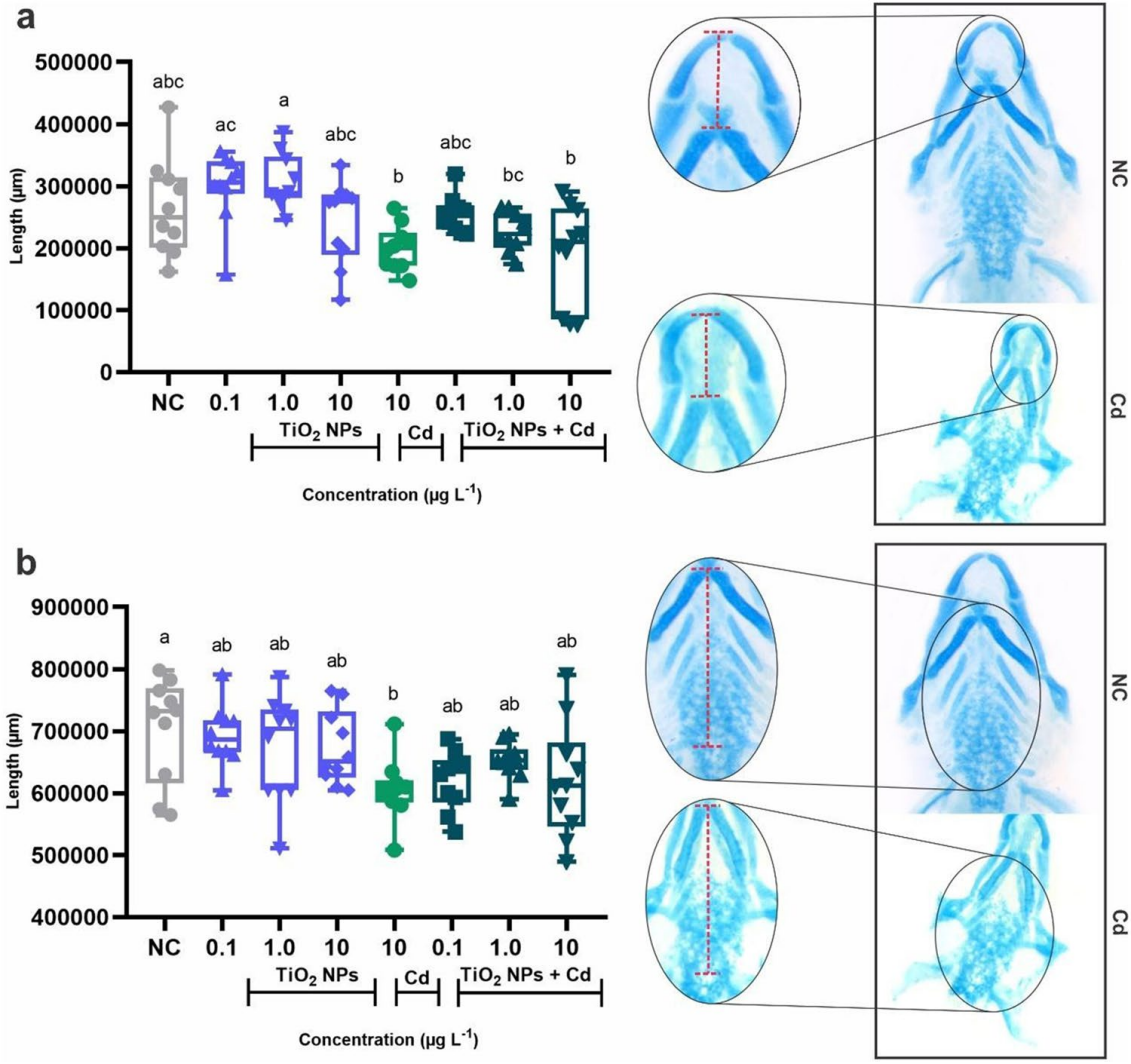
Changes in the chondrocranium of zebrafish larvae after exposure to titanium dioxide nanoparticles (TiO_2_ NPs) and cadmium (Cd), single or combined, after 168 h under semi-static conditions. **a** Length between the MK cartilage and the CH; **b** length between the CH cartilage and the posterior end of the head. Different letters represent statistically different groups (*p* < 0.05)

**Fig. 5:**
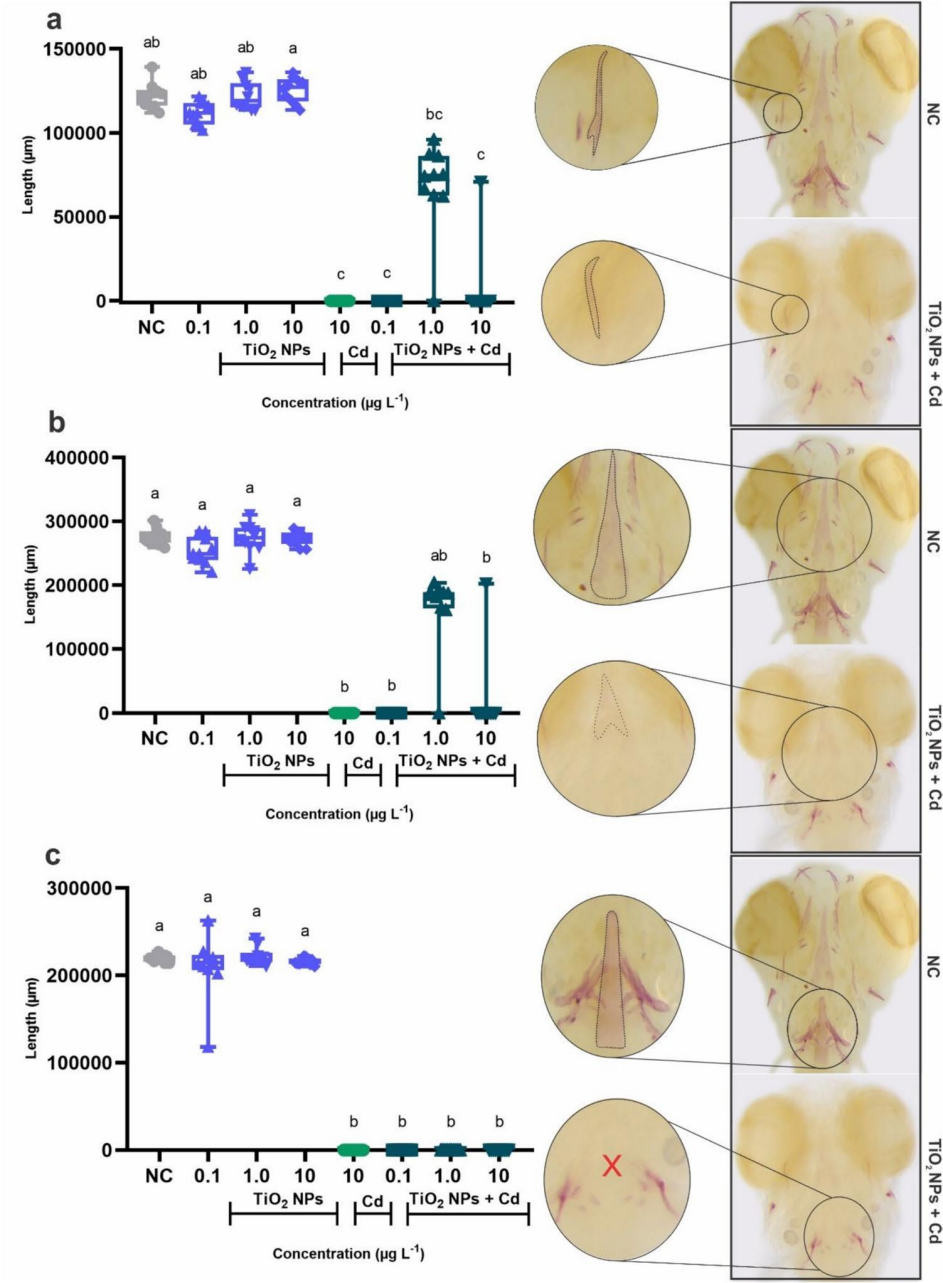
Length of the branchiostegal ray bone 2 (**a**), parasphenoid bone (**b**), and ossified sheath of the notochord (**c**) of zebrafish larvae after exposure to titanium dioxide nanoparticles (TiO_2_ NPs) and cadmium (Cd), single or combined, after 168 h under semi-static conditions. Different letters represent statistically different groups (*p* < 0.05)

**Fig. 6:**
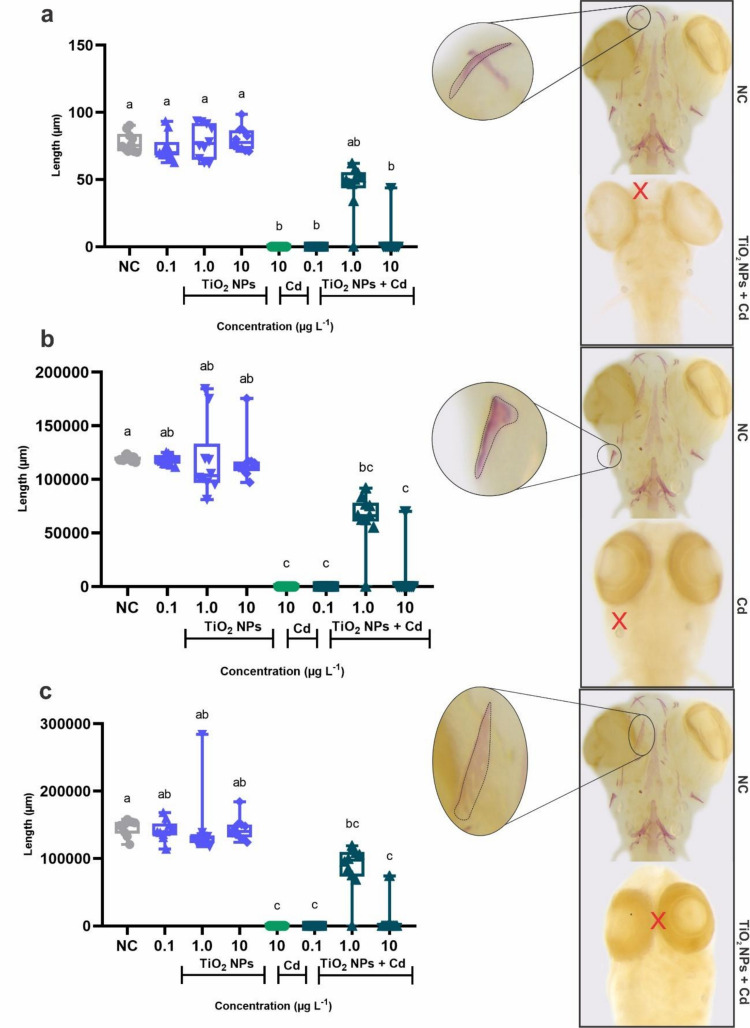
Length of the dental bones (**a**), operculum (**b**), and length of the entopterygoid (**c**) of zebrafish larvae after exposure to titanium dioxide nanoparticles (TiO_2_ NPs) and cadmium (Cd), single or combined, after 168 h under semi-static conditions. Different letters represent statistically different groups (*p* < 0.05)

Results on chondrocranium formation (Figs. 2, 3, and 4) and calcification of craniofacial bones (Figs. 5 and 6) provided clear evidence of a chondrotoxic and anti-osteogenic effect of single Cd and combined with TiO_2_ NPs. Reports of Cd toxicity in the skeletal formation of zebrafish have already been documented (Tarasco et al. 2019); however, the current study is a pioneer in evaluating the skeletal toxicity induced by single TiO_2_ NPs or combined with Cd.

#### Changes to the chondrocranium in zebrafish larvae

The exposure to single TiO_2_ NPs did not induce changes in the chondrocranium of the zebrafish larvae. However, the combined TiO_2_ NPs and Cd reduced (53.7 to 75.3%) the distance between the branches of the Meckel cartilage (MK) at all concentrations tested (*p* < 0.01; Fig. 2a). Similarly, the distance between branches of the ceratohyal cartilage (CH) was reduced (42.5 to 74.9%) when co-exposed to TiO_2_ NPs and Cd (*p* < 0.001), also when exposed to single Cd (*p* < 0.01; Fig. 2b).

TiO_2_ NPs and Cd mixture reduced the angle between the MK cartilage and palatoquadrate (PQ) cartilage joint, only at the highest concentration (10 µg L^−1^) (*p* < 0.01; Fig. 3a). Similarly, the exposure to single Cd and the highest concentrations (1.0 and 10 µg L^−1^) combined with TiO_2_ NPs reduced the angle between the PQ and CH cartilages (Fig. 3b). In both measurements, single TiO_2_ NPs did not cause any skeletal changes. Furthermore, there were no significant changes in any of the groups evaluated in terms of differences in the length between the MK and CH cartilage (Fig. 4a). Only Cd reduced the length between the CH cartilage and the posterior end of the head (*p* < 0.05; Fig. 4b).

As previously observed in the cartilaginous structures of the chondrocranium of zebrafish larvae, the interaction between TiO_2_ NPs and Cd resulted in more pronounced negative effects when compared to single exposure to TiO_2_ NPs. Such alterations can significantly affect the morphology of an animal’s head, potentially compromising its chances of survival. The chondrocranium, a transitional cartilaginous structure that makes up the skull of fish, plays a crucial role in the formation of the larval head, providing essential structural support for functions such as breathing and feeding, as well as playing a vital role in protecting sensory organs (Kimmel et al. 2001). During the development of the chondrocranium, the MK and CH cartilages of the pharyngeal skeleton are major structural support bars for mandible formation (Schilling et al. 1996). Abnormal development, such as the changes in chondrocranium structures presented in this study, after direct exposure to single Cd and combined with TiO_2_ NPs, could result in smaller skulls and affect behavior during the larval stage, especially in the feeding process of zebrafish (Cohen & Smith 2014). In addition, other changes in the conformation of the chondrocranium have already been reported, such as a decrease in the angle of the CH cartilage after direct exposure to Cd (3.7 to 685 µg L^−1^) (Wu et al. 2018).

In another study, which investigated the effects of 4-day parental exposure to CdSe/ZnS QDs on the skeletal development of the F1 generation of *G. rarus*, considering the potential Cd-related toxicity of QDs, adverse effects on growth and development were observed. Specifically, there was a significant impact on the cartilage development of F1 larvae by affecting the chondrocytes and altering the length and angle of the MK, CH, and PQ cartilage structures of the pharyngeal chondrocranium skeleton (Chen et al. 2022b). Changes in the length of these cartilages consequently alter the angles of the cartilage bars and modify the size of the head, which is certainly a good indicator for checking the degree of chondrogenesis (Mork & Crump 2015), as demonstrated in this study.

The changes observed in the chondrocranium, associated with exposure to Cd, both alone and in co-exposure with TiO₂ NPs, such as modification of the distance between the branches of the MK and CH cartilages, which will consequently reduce the values of their angles and the consequent modification of the length of the chondrocranium, may be related to a possible decrease in calcium levels (Ca^2^⁺). According to the literature, this reduction may be caused by the inhibition of Ca^2^⁺ absorption by Cd, directly affecting cartilage development (Matta et al. 2015; Uzieliene et al. 2018; Wong & Wong 2000). For example, *Oreochromis mossambicus* exposed to Cd (40 × 10^3^, 80 × 10^3^, and 160 × 10^3^ µg L^−1^) for 7 days showed a decrease in the activities of alkaline Ca^2+^-ATPases of gills, indicating a reduction in Ca^2+^ transport capacity per unit cell. This phenomenon can result in hypocalcaemia in fish (Wong & Wong 2000). Ca^2+^ is essential for chondrogenesis, as it is a signalling factor for chondrogenic differentiation from mesenchymal cells (Matta & Zakany 2013; Steward et al. 2014; Uzieliene et al. 2018). Intracellular Ca^2+^ participates in various signalling pathways, including several protein kinase systems. This participation can allow for distinct gene expression profiles through the differential activation of key transcription factors, resulting in chondrogenic differentiation (Mobasheri et al. 2019). In addition, chondrocytes have a variety of ion channels, including large, small-conductance Ca^2+^-activated potassium channels. These channels are crucial for the efficient renewal of the extracellular matrix and the maintenance of homeostasis (Matta et al. 2015). Therefore, it is important to deepen our understanding of the aetiology of Cd toxicity associated with TiO_2_ NPs in chondrocranium cartilage development, and zebrafish has proven to be an appropriate model system to investigate skeletal toxicity.

Similarly, several researchers have focused their analyses on the interactive effects between TiO_2_ NPs and Cd in aquatic animal models, highlighting the cumulative results of Cd potentiated by TiO_2_ NPs, a phenomenon known as the “Trojan Horse”. The bioaccumulation of Cd (100 to 200 µg L^−1^) in adult zebrafish was identified after combined with TiO_2_ NPs (4 × 10^3^ µg L^−1^) over 96 h (Huang et al. 2021). The association between TiO_2_ NPs (1.0 and 10 µg L^−1^) and Cd (10 µg L^−1^), at environmentally relevant concentrations, also induced more adverse effects (cardiotoxicity, morphological, and morphometric changes) in zebrafish embryos and larvae compared to single TiO_2_ NPs (Mamboungou et al. 2022). Similar results can be seen in the invertebrate groups, such as the species *Lytechinus variegatus* co-exposed to Cd (from 100 to 506 μg L^−1^ for 96 h) and for *D. magna* (from 10 to 640 μg L^−1^ for 48 h) with TiO_2_ NPs (2 × 10^3^ µg L^−1^) (Hartmann et al. 2012), and *Corbicula fluminea* exposed to higher concentrations of TiO_2_ NPs (3% of total sediment mass) and Cd (10.2 μg g^−1^, for 14 days) (Fan et al. 2018), confirming the risk of these combined to the health of aquatic organisms.

#### Craniofacial bone changes in zebrafish larvae

Single Cd and combined with TiO_2_ NPs induced osteotoxic effects in zebrafish larvae in all the parameters analyzed. For example, there was a 43.9 to 94.2% reduction in the size of the branchiostegal ray bone 2 (Br2) as a result of combined Cd and TiO_2_ NPs (1.0 to 10 µg L^−1^), as well the absence of formation of Br2 in larvae exposed to single Cd and TiO_2_ NPs at the lowest concentration (0.1 µg L^−1^) associated with Cd (Fig. 5a). Likewise, the parasphenoid bone (PH) was not calcified when exposed to single Cd or when Cd was combined with TiO_2_ NPs at the lowest concentration (0.1 µg L^−1^), but at higher concentrations of TiO_2_ NPs (1.0 to 10 µg L^−1^) co-exposed with Cd, there was a reduction of 40.0 to 92.7% in the size of the PH (*p* < 0.05; Fig. 5b). As for the ossified sheath of the notochord (NO), the treatments involving the combined TiO_2_ NPs (0.1, 1.0, and 10 µg L^−1^) associated with Cd completely inhibited formation, as did the treatments exposed to single Cd (Fig. 5c).

Single Cd was responsible for inhibiting bone development in the dental (DT), operculum (OP), and entopterygoid (EN) bones (*p* < 0.01; Fig. 6A, B, and C). The same results were also observed in the larvae co-exposed to TiO_2_ NPs and Cd, resulting in complete inhibition of the DT, OP, and EN bones (Fig. 6A, B, and C). On the other hand, as a result of the association between TiO_2_ NPs (1.0 and 10 µg L^−1^) and Cd (10 µg L^−1^), DT, OP, and EN bones were formed, but showed an equally significant reduction concerning their respective sizes at the presented stage (*p* < 0.001; Fig. 6A, B, and C).

As seen above, the metal Cd completely inhibited the ossification process of the Br2, PH, NO, DT, OP, and EN bones in zebrafish larvae after 168 h of exposure. With single exposure to TiO_2_ NPs, there was no inhibition or reduction in the degree of ossification of craniofacial structures (Figs. 5 and 6). On the other hand, the Cd-TiO_2_ NP interaction caused inhibition or a large reduction in bone mineralization at concentrations of 0.1 and 10 µg L^−1^ of TiO_2_ NPs. However, at the intermediate concentration of 1.0 µg L^−1^ of TiO_2_ NPs, there was less reduction in the calcification. With this, it can be inferred that the Cd-TiO_2_ NPs combined showed concentration-dependent toxicity, where there may have been a negative Trojan Horse effect at the intermediate concentration of TiO_2_ NPs.

As for Cd, this result is consistent with previous findings in the literature. *G. affinis* juveniles after exposure to Cd (400 µg L^−1^) showed spinal deformities, such as kyphosis, lordosis, scoliosis, bifurcated neural and haemal spines, fractured, and malformed haemal spines (Sassi et al. 2010). In larvae of *Sparus aurata*, exposure to Cd (5 × 10^3^ and 10 × 10^3^ µg L^−1^) affected osteocalcin gene expression (e.g., *mt*, *tnfα*, *hsp70*, and *oc*) and interrupted bone mineralization (Sassi et al. 2013). Fish scales have also been analyzed to show the osteotoxicity of Cd. This was seen when observing deformations in the scales of *C. carpio*, induced by different Cd concentrations (14.5, 29, 43.5, 58 µg L^−1^) (Rishi & Jain 1998) and 250 and 2500 µg g^−1^ (Yoshitomi et al. 1998) and in *Carassius auratus*, by influencing osteoclastic activities under acute exposure and inhibiting osteoblastic activities under prolonged Cd exposure (112,411 × 10^–12^ to 112,411 × 10^–7^ µg L^−1^) (Suzuki et al. 2004).

The inhibitory effect of Cd on the in vitro calcification of MC3T3-E1 cells was previously identified by Miyahara et al. (1988). The authors suggested that Cd had the potential to inhibit both the differentiation of osteoblasts and their cellular function, resulting in a reduction in the physicochemical deposition of minerals. Reinforcing this hypothesis, Cd decreased the expression of marker genes for the maturation and function of osteoblasts in zebrafish larvae at 6 days post-fertilization (dpf) (10 µg L^−1^), causing a reduction in opercular bone growth in 6 dpf larvae (5 and 10 µg L^−1^ of Cd) and an increase in the incidence of skeletal deformities in 20 dpf larvae (1 and 5 µg L^−1^ of Cd). In addition, the effect of Cd (0.3 and 3.16 µg L^−1^) on caudal fin bone regeneration in adult zebrafish was investigated after exposure for 5 days, observing changes in the length and bone rigidity of the newly formed fin (Tarasco et al. 2019).

Although these data indicate the skeletal toxicity of Cd in fish, the underlying mechanisms are still not fully understood, and there are several studies demonstrating different signalling pathways for the inhibition or reduction of bone mineralization (Luo et al. 2021; Pi et al. 2019; Rodríguez & Mandalunis 2016; Yang et al. 2016). One or several mechanisms may be involved directly or indirectly impacting bone development or the quality of the bone already formed. With this in mind, many researchers have dedicated themselves to studying the bone toxicity of Cd in different animal models. It has been found that Cd can inhibit the differentiation of mesenchymal stem cells (MSCs) into osteoblasts and cause apoptosis, both in vivo and in vitro (Ma et al. 2021b, 2021a). In addition, Cd-induced mitochondrial dysfunction may be one of the main reasons for MSCs death (Wu et al. 2019). MSCs play an essential role in maintaining the quantity of osteoblastic cells, which effectively regulate the metabolism and remodeling of bone tissue (Mehranjani & Mosavi 2011). MSC dysfunction is one of the main causes of bone damage induced by Cd (Ma et al. 2021a). In another way, Cd can induce osteoblastic damage and oxidative stress, causing DNA damage, mitochondrial dysfunction, and endoplasmic reticulum stress, resulting in apoptosis (Brama et al. 2012; Liu et al. 2014; Oliveira et al. 2014; Smith et al. 2009). It is worth emphasizing that the mechanisms by which Cd induces apoptosis may be diverse (Coonse et al. 2007; Liu et al. 2016; Zhao et al. 2015). However, Cd-induced osteoblast dysfunction not only modifies morphological structure but also manifests severe genotoxicity. It has also been shown that Cd (56 × 10^4^ µg L^−1^) can affect osteoclast activation and promote bone resorption (Suzuki et al. 1990), stimulation of multinucleated osteoclast formation (Miyahara et al. 1992) and increased number of osteoclasts and resorption area at low Cd concentrations (1–11 µg L^−1^) (Wilson et al. 1996). These changes can result in bone loss, making them more fragile and prone to developing osteoporosis (Ma et al. 2021b).

#### Condition index of changes in the skeletal system

**Fig. 7:**
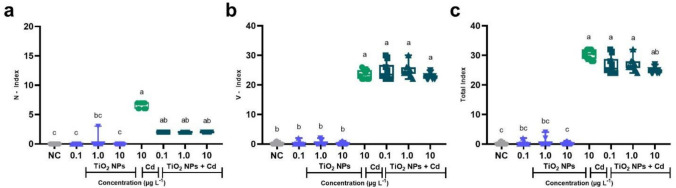
Condition index of changes in the skeletal system in the skull of zebrafish larvae after exposure to titanium dioxide nanoparticles (TiO_2_ NPs) and cadmium (Cd), single or combined, after 168 h under semi-static conditions. **a** Neurocranium index (N-index); **b** viscerocranium index (V-Index), and **c** total index. Different letters represent statistically different groups (*p* < 0.05)

The neurocranium and viscerocranium were significantly altered in all groups exposed to single Cd and combined with TiO_2_ NPs (Fig. 7). The viscerocranium was more affected by exposure to single Cd and associated with TiO_2_ NPs than the neurocranium (Fig. 7A and B). In addition, the total skull alteration index showed the same results as those mentioned above (Fig. 7C). As with the other results related to TiO_2_ NPs, there were no significant effects in the neurocranium and viscerocranium.

### Locomotor behavior analysis

**Fig. 8:**
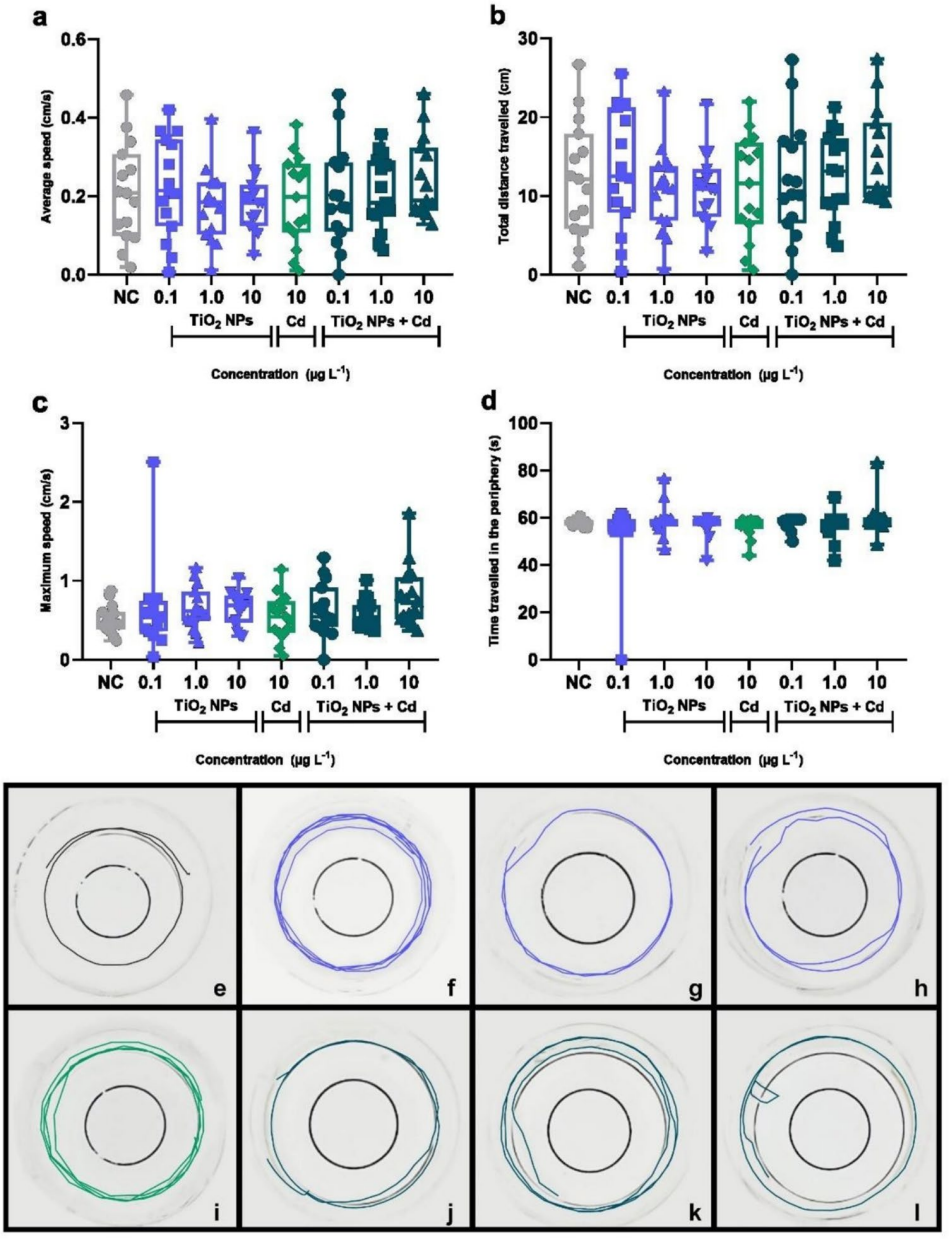
Locomotion behavior in zebrafish larvae after exposure to TiO_2_ NPs and Cd concentrations, both single and combined for 144 h. **A** Average speed, **B** total distance travelled, **C** maximum speed, and **D** time travelled in the periphery. Board with the total distance travelled: **E** negative control, **F** 0.1 µg L^−1^ of TiO_2_ NPs, **G** 1.0 µg L^−1^ of TiO_2_ NPs, **H** 10 µg L^−1^ of TiO_2_ NPs, **I** 10 µg L^−1^ of Cd, **J** 0.1 µg L^−1^ of TiO_2_ NPs and 10 µg L^−1^ of Cd, **K** 1.0 µg L^−1^ of TiO_2_ NPs and 10 µg L^−1^ of Cd, and **L**10 µg L^−1^ of TiO_2_ NPs and 10 µg L^−1^ of Cd

 The exposure to TiO_2_ NPs and Cd, single and combined, for 144 h did not change the locomotor activity (average speed, maximum speed, total distance traveled, and time traveled in the periphery) in zebrafish larvae (*p* > 0.05; Fig. 8). Corroborating these results, zebrafish larvae exposed to Cd (7.67 μg L^−1^) showed an average speed similar to that observed in the control group (Han et al. 2019). Similarly, combined TiO_2_ NPs (100 μg L^−1^) and Pb (10 and 20 μg L^−1^) did not result in significant differences compared to the control group in the evaluations of average speed and distance traveled (Hu et al. 2019).

Although the data from this study do not demonstrate changes in locomotor behavior, previous studies have reported adverse effects in zebrafish larvae exposed to Cd (11 µg L^−1^) for 5 dpf, such as a significant reduction in the distance traveled and average swimming speed (Tu et al. 2017). Sloman et al. (2003) observed negative effects on the competitive behavior of rainbow trout (*Oncorhynchus mykiss*) exposed to Cd (2 μg L^−1^) for 24 h. Also, zebrafish larvae exposed to TiO_2_ NPs (1000 μg L^−1^) and the insecticide cypermethrin (2 L^−1^), single and combined, showed a reduction in the maximum and average swimming speed of the larvae; however, combined, there was a more pronounced reduction (Li et al. 2018), suggesting that TiO_2_ NPs may act as a vehicle for other chemicals (Naasz et al. 2018). These findings highlight the importance of considering the potential interactive effects of different chemicals in the aquatic environment and the possible impacts on aquatic fauna.

As observed, environmentally relevant concentrations of TiO_2_ NPs did not interfere with the skeletal formation of the animal, nor did they alter locomotor behavior. However, although exposure to single Cd and combined with TiO_2_ NPs resulted in skeletal changes, no behavioral changes were observed after 144 h of exposure. This may have occurred because, at this larval stage, cartilage is predominantly present in the craniofacial skeleton of the animal, and it is a temporary structure that precedes the deposition of calcium for ossification (Matta & Zakany 2013). In a way, at this stage of development, the chondrocranium may provide some stability in terms of physical fitness for swimming. Furthermore, the larvae may possess neuromuscular compensatory mechanisms that aid locomotion, and their muscular structure assists in swimming movements (Dou et al. 2008).

## Conclusion

The current study showed that Cd, both in isolation and in interaction with TiO_2_ NPs, induced skeletal toxicity in zebrafish larvae. Although single TiO_2_ NPs exposure did not show skeletal toxicity to the larvae, the combination of Cd and TiO_2_ NPs caused significant alterations. The results highlight the toxicity of Cd, as it caused skeletal malformations in the exposed larvae, both single and in combination. However, it is observed that, when combined with TiO_2_ NPs at higher concentrations, we can infer that there was concentration-dependent toxicity, with a possible negative “Trojan Horse” effect. TiO_2_ NPs may have played a role in reducing Cd toxicity in certain cases, such as in the bone malformations observed at 1.0 + 10 and 10 + 10 µg L^−1^ concentrations. However, for more robust confirmation, an analysis of the crystallization of TiO₂ NPs would be required, it is therefore important that future studies include a full crystallographic characterization, so as to enable meaningful comparisons and a risk assessment. Regarding the locomotor behavior of the animal, no significant changes were observed in the zebrafish larvae exposed to Cd and TiO_2_ NPs, single or in combination. The results highlight the importance of further studies on the toxicity of the interaction between NMs, such as NPs and metals, for aquatic organisms after chronic exposures or using endemic species.

This study showed that zebrafish is an excellent model for nanotoxicology studies, especially for analyses of skeletal morphology, due to its biological characteristics that facilitate the application of well-established techniques for skeletal assessment. Nevertheless, it is essential to conduct further studies with different fish species, particularly Brazilian endemic species, in order to broaden our understanding of the impacts arising from the interaction between these contaminants. Such investigations are essential to inform strategies for the monitoring, conservation, and preservation of aquatic fauna.

## Data Availability

Data will be made available on request.

## References

[CR1] Abd-Elhakim YM, Hashem MM, Abo-EL-Sooud K, Hassan BA, Elbohi KM, Al-Sagheer AA (2021) Effects of co-exposure of nanoparticles and metals on different organisms: a review. Toxics 9:284. 10.3390/toxics9110284 34822675 10.3390/toxics9110284 PMC8623643

[CR2] Avdesh A, Chen M, Martin-Iverson MT, Mondal A, Ong D, Rainey-Smith S, Taddei K, Lardelli M, Groth DM, Verdile G, Martins RN (2012) Regular care and maintenance of a Zebrafish (*Danio rerio*) laboratory: an introduction. J Vis Exp 69. 10.3791/4196 10.3791/4196 PMC3916945 23183629

[CR3] Bar-Ilan O, Chuang CC, Schwahn DJ, Yang S, Joshi S, Pedersen JA, Hamers RJ, Peterson RE, Heideman W (2013) TiO_2_ nanoparticle exposure and illumination during zebrafish development: mortality at parts per billion concentrations. Environ Sci Technol 47(9):4726–4733. 10.1021/es304514r 23510150 10.1021/es304514r

[CR4] Barnard AS, Curtiss LA (2005) Prediction of TiO2 nanoparticle phase and shape Transitions Controlled by Surface Chemistry. Nano Letters 5(7):1261–1266. 10.1021/nl050355m 16178221 10.1021/nl050355m

[CR5] Binelli A, Del Giacco L, Santo N, Bini L, Magni S, Parolini M, Madaschi L, Ghilardi A, Maggioni D, Ascagni M, Armini A, Prosperi L, Landi C, La Porta C, Della Torre C (2017) Carbon nanopowder acts as a Trojan-Horse for benzo(α)pyrene in *Danio Rerio* embryos. Nanotoxicology 11(3):371–381. 10.1080/17435390.2017.1306130 28285553 10.1080/17435390.2017.1306130

[CR6] Bird NC, Mabee PM (2003) Developmental morphology of the axial skeleton of the zebrafish, *Danio rerio* (Ostariophysi: Cyprinidae). Dev Dyn 228(3):337–357. 10.1002/dvdy.10387 10.1002/dvdy.10387 14579374

[CR7] Brama M, Politi L, Santini P, Migliaccio S, Scandurra R (2012) Cadmium-induced apoptosis and necrosis in human osteoblasts: role of caspases andmitogen-activated protein kinases pathways. J Endocrinol Invest 35(2):198–208. 10.3275/7801 21697648 10.3275/7801

[CR8] Brasil (2005) Resolução CONAMA n° 357, de 17 de Março de 2005 (Retificada). Conselho Nacional Do Meio Ambiente, 204, 36. https://conama.mma.gov.br/?option=com_sisconama&task=arquivo.download&id=450. Accessed 3 Jul 2026

[CR9] Chang HH, Cheng TJ, Huang CP, Wang GS (2017) Characterization of titanium dioxide nanoparticle removal in simulated drinking water treatment processes. Sci Total Environ 601–602(17):886–894. 10.1016/j.scitotenv.2017.05.228 10.1016/j.scitotenv.2017.05.228 28582734

[CR10] Chen A, Deng H, Song X, Liu X, Chai L (2022) Effects of separate and combined exposure of cadmium and lead on the endochondral ossification in *Bufo gargarizans*. Environ Toxicol Chem 41(5):1228–1245. 10.1002/etc.5296 35040517 10.1002/etc.5296

[CR11] Chen J, Chen H, Wu Y, Meng J, Jin L (2022) Parental exposure to CdSe/ZnS QDs affects cartilage development in rare minnow (*Gobiocypris rarus*) offspring. Comparative Biochemistry and Physiology Part - C: Toxicology and Pharmacology 256(1):109304. 10.1016/j.cbpc.2022.109304 35257888 10.1016/j.cbpc.2022.109304

[CR12] Chen P, Chen B, He M, Zhou Y, Lei L, Han J, Zhou B, Hu L, Hu B (2025) Nanoplastics and nano-ZnO facilitate Cd accumulation in zebrafish larvae via a distinct pathway: revelation by LA-ICP-MS imaging. Chin Chem Lett 36(2):109908. 10.1016/j.cclet.2024.109908

[CR13] Choong G, Liu Y, Templeton DM (2014) Interplay of calcium and cadmium in mediating cadmium toxicity. Chem Biol Interact 211(1):54–65. 10.1016/j.cbi.2014.01.007 24463198 10.1016/j.cbi.2014.01.007

[CR14] Cohen A, Smith Y (2014) Estrogen regulation of microRNAs, target genes, and microRNA expression associated with vitellogenesis in the zebrafish. Zebrafish 11(5):462–478. 10.1089/zeb.2013.0873 23767875 10.1089/zeb.2013.0873 PMC4172469

[CR15] Coonse KG, Coonts AJ, Morrison EV, Heggland SJ (2007) Cadmium induces apoptosis in the human osteoblast-like cell line Saos-2. Journal of Toxicology and Environmental Health - Part a: Current Issues 70(7):575–581. 10.1080/15287390600882663 10.1080/15287390600882663 17365611

[CR16] Dammski AP, Müller BR, Gaya C, Regonato D (2011) Zebrafish: Manual de criação em biotério (1st edn). Universidade Federal do Paraná

[CR17] Dedman CJ, King AM, Christie-Oleza JA, Davies GL (2021) Environmentally relevant concentrations of titanium dioxide nanoparticles pose negligible risk to marine microbes. Environ Sci Nano 8(5):1236–1255. 10.1039/d0en00883d 34046180 10.1039/d0en00883d PMC8136324

[CR18] Della Torre C, Balbi T, Grassi G, Frenzilli G, Bernardeschi M, Smerilli A, Guidi P, Canesi L, Nigro M, Monaci F, Scarcelli V, Rocco L, Focardi S, Monopoli M, Corsi I (2015) Titanium dioxide nanoparticles modulate the toxicological response to cadmium in the gills of *Mytilus galloprovincialis*. J Hazard Mater 297:92–100. 10.1016/j.jhazmat.2015.04.072 25956639 10.1016/j.jhazmat.2015.04.072

[CR19] Dingerkus G, Uhler LD (1977) Enzyme clearing of alcian blue stained whole small vertebrates for demonstration of cartilage. Biotech Histochem 52(4):229–232. 10.3109/10520297709116780 10.3109/10520297709116780 71769

[CR20] Disner GR, Guiloski IC, Klingelfus T, Silva LFO, Lirola JR, de Assis HCS, Cestari MM (2017) Co-exposure effects of titanium dioxide nanoparticles and metals on antioxidant systems and DNA in the fish *Hoplias intermedius*. Ecotoxicology and Environmental Contamination 12(1):75–84. 10.5132/eec.2017.01.10

[CR21] Dou Y, Andersson-Lendahl M, Arner A (2008) Structure and function of skeletal muscle in zebrafish early larvae. J Gen Physiol 131(5):445–453. 10.1085/jgp.200809982 18443359 10.1085/jgp.200809982 PMC2346565

[CR22] Dover G (2000) How genomic and developmental dynamics affect evolutionary processes. BioEssays 22(12):1153–1159. 10.1002/1521-1878(200012)22:12<1153::AID-BIES13>3.0.CO;2-0 11084631 10.1002/1521-1878(200012)22:12<1153::AID-BIES13>3.0.CO;2-0

[CR23] Fan X, Wang P, Wang C, Hu B, Wang X (2017) Lead accumulation (adsorption and absorption) by the freshwater bivalve Corbicula fluminea in sediments contaminated by TiO2 nanoparticles. Environ Pollut 712–721. 10.1016/j.envpol.2017.08.080 10.1016/j.envpol.2017.08.080 28850939

[CR24] Fan X, Wang C, Wang P, Hu B, Wang X (2018) TiO_2_ nanoparticles in sediments: effect on the bioavailability of heavy metals in the freshwater bivalve *Corbicula fluminea*. J Hazard Mater 342:41–50. 10.1016/j.jhazmat.2017.07.041 28822248 10.1016/j.jhazmat.2017.07.041

[CR25] Figueirêdo LP, Cirqueira F, Sousa BLC, Mamboungou J, Rocha TL (2025) Developmental toxicity of formulated insecticide mixture containing imidacloprid and beta-cyfluthrin in fish: Insights using zebrafish. Chemosphere 377:144314. 10.1016/j.chemosphere.2025.144314 10.1016/j.chemosphere.2025.144314 40132347

[CR26] Genchi GSS, Graziantono L, Carocci A, Catalano A (2020) The effects of toxicity. Int J Environ Res Public Health 17(Cd):1–24

[CR27] Gu J, Guo M, Huang C, Wang X, Zhu Y, Wang L, Wang Z, Zhou L, Fan D, Shi L, Ji G (2021) Titanium dioxide nanoparticle affects motor behavior, neurodevelopment and axonal growth in zebrafish (*Danio rerio*) larvae. Sci Total Environ 754:142315. 10.1016/j.scitotenv.2020.142315 33254858 10.1016/j.scitotenv.2020.142315

[CR28] Han J, Liu K, Wang R, Zhang Y, Zhou B (2019) Exposure to cadmium causes inhibition of otolith development and behavioral impairment in zebrafish larvae. Aquat Toxicol 214(June). 10.1016/j.aquatox.2019.105236 10.1016/j.aquatox.2019.105236 31260825

[CR29] Hartmann NB, Legros S, Von der Kammer F, Hofmann T, Baun A (2012) The potential of TiO_2_ nanoparticles as carriers for cadmium uptake in *Lumbriculus variegatus* and *Daphnia magna*. Aquat Toxicol 118–119:1–8. 10.1016/j.aquatox.2012.03.008 10.1016/j.aquatox.2012.03.008 22494961

[CR30] Hu X, Chen Q, Jiang L, Yu Z, Jiang D, Yin D (2011) Combined effects of titanium dioxide and humic acid on the bioaccumulation of cadmium in Zebrafish. Environ Pollut 159(5):1151–1158. 10.1016/j.envpol.2011.02.011 21376439 10.1016/j.envpol.2011.02.011

[CR31] Hu S, Han J, Yang L, Li S, Guo Y, Zhou B, Wu H (2019) Impact of co-exposure to titanium dioxide nanoparticles and Pb on zebrafish embryos. Chemosphere 233:579–589. 10.1016/j.chemosphere.2019.06.009 31195263 10.1016/j.chemosphere.2019.06.009

[CR32] Huang B, Cui Y-Q, Guo W-B, Yang L, Miao AJ (2021) Regulation of cadmium bioaccumulation in zebrafish by the aggregation state of TiO_2_ nanoparticles. J Hazard Mater 126510. 10.1016/j.jhazmat.2021.126510 10.1016/j.jhazmat.2021.126510 34216965

[CR33] Iqbal J, Shah MH, Akhter G (2013) Characterization, source apportionment and health risk assessment of trace metals in freshwater Rawal Lake, Pakistan. Journal of Geochemical Exploration 125:94–101. 10.1016/j.gexplo.2012.11.009

[CR34] ISO (1996) Water quality determination of the acute lethal toxicity of substances to a freshwater fish[*Brachydanio rerio* Hamilton Buchanan (Teleostei, Cyprinidae)]. Part 3. Flow-through method. International Organization for Standardization. https://www.iso.org/standard/14030.html?__cf_chl_f_tk=aEnjqdiqxMUlmi_YPBQ_oJyHvWjx9BIVxdVf10eb_5o-1783086937-1.0.1.1-hcSp4DIcPdWLw_ZZNCOjdISwFebp.CoY1Rc_yEaE470. Accessed 3 Jul 2026

[CR35] Kimmel CB, Ballard WW, Kimmel SR, Ullmann B, Schilling TF (1995) Stages of embryonic development of the zebrafish. Dev Dyn 203(3):253–310. 10.1002/aja.1002030302 8589427 10.1002/aja.1002030302

[CR36] Kimmel CB, Miller CT, Moens CB (2001) Specification and morphogenesis of the zebrafish larval head skeleton. Dev Biol 233(2):239–257. 10.1006/dbio.2001.0201 11336493 10.1006/dbio.2001.0201

[CR37] Lacave JM, Bilbao E, Gilliland D, Mura F, Dini L, Cajaraville MP, Orbea A (2020) Bioaccumulation, cellular and molecular effects in adult zebrafish after exposure to cadmium sulphide nanoparticles and to ionic cadmium. Chemosphere 238. 10.1016/j.chemosphere.2019.124588 10.1016/j.chemosphere.2019.124588 31545210

[CR38] Lammer E, Carr GJ, Wendler K, Rawlings JM, Belanger SE, Braunbeck T (2009) Is the fish embryo toxicity test (FET) with the zebrafish (*Danio rerio*) a potential alternative for the fish acute toxicity test? Comparative Biochemistry and Physiology - C Toxicology and Pharmacology 149(2):196–209. 10.1016/j.cbpc.2008.11.006 19095081 10.1016/j.cbpc.2008.11.006

[CR39] Li M, Wu Q, Wang Q, Xiang D, Zhu G (2018) Effect of titanium dioxide nanoparticles on the bioavailability and neurotoxicity of cypermethrin in zebrafish larvae. Aquat Toxicol 199(January):212–219. 10.1016/j.aquatox.2018.03.022 29656190 10.1016/j.aquatox.2018.03.022

[CR40] Liu G, Zou H, Luo T, Long M, Bian J, Liu X, Gu J, Yuan Y, Song R, Wang Y, Zhu J, Liu Z (2016) Caspase-dependent and caspase-independent pathways are involved in cadmium-induced apoptosis in primary rat proximal tubular cell culture. PLoS ONE 11(11):1–17. 10.1371/journal.pone.0166823 10.1371/journal.pone.0166823 PMC5115828 27861627

[CR41] Liu W, Zhao H, Wang Y, Jiang C, Xia P, Gu J, Liu X, Bian J, Yuan Y, Liu Z (2014) Calcium-calmodulin signaling elicits mitochondrial dysfunction and the release of cytochrome c during cadmium-induced apoptosis in primary osteoblasts. Toxicol Lett 224(1):1–6. 10.1016/j.toxlet.2013.10.009 24144892 10.1016/j.toxlet.2013.10.009

[CR42] Luo H, Gu R, Ouyang H, Wang L, Shi S, Ji Y, Bao B, Liao G, Xu B (2021) Cadmium exposure induces osteoporosis through cellular senescence, associated with activation of NF-κB pathway and mitochondrial dysfunction. Environmental Pollution 290(December 2020):118043. 10.1016/j.envpol.2021.118043 34479166 10.1016/j.envpol.2021.118043

[CR43] Ma Y, Ran D, Cao Y, Zhao H, Song R, Zou H, Gu J, Yuan Y, Bian J, Zhu J, Liu Z (2021b) The effect of P2X7 on cadmium-induced osteoporosis in mice. Journal of Hazardous Materials 405(July 2020):124251. 10.1016/j.jhazmat.2020.124251 33168313 10.1016/j.jhazmat.2020.124251

[CR44] Ma Y, Ran D, Shi X, Zhao H, Liu Z (2021) Cadmium toxicity: a role in bone cell function and teeth development. Sci Total Environ 769:144646. 10.1016/j.scitotenv.2020.144646 33485206 10.1016/j.scitotenv.2020.144646

[CR45] MacCuspie RI, Rogers K, Patra M, Suo Z, Allen AJ, Martin MN, Hackley VA (2011) Challenges for physical characterization of silver nanoparticles under pristine and environmentally relevant conditions. J Environ Monit 13(5):1212–1226. 10.1039/c1em10024f 21416095 10.1039/c1em10024f

[CR46] Mahecha GAB, de Oliveira CA (1994) A modified technique of diaphanisation and differential staining of cartilage and bone in small vertebrates. Rev Bras Ciênc Morfol 11(2):204

[CR47] Mamboungou J, Canedo A, Qualhato G, Rocha TL, Vieira LG (2022). Environmental risk of titanium dioxide nanoparticle and cadmium mixture: developmental toxicity assessment in zebrafish (*Danio rerio*). J Nanopart Res 24(9). 10.1007/s11051-022-05561-w

[CR48] Matta C, Zakany R (2013) Calcium signalling in chondrogenesis: implications for cartilage repair. Front Biosci 55:305–324. 10.2741/s374 10.2741/s374 23277053

[CR49] Matta C, Zákány R, Mobasheri A (2015) Voltage-dependent calcium channels in chondrocytes: roles in health and disease. Curr Rheumatol Rep 17(7). 10.1007/s11926-015-0521-4 10.1007/s11926-015-0521-4 25980668

[CR50] Mehranjani M, Mosavi M (2011) Cadmium chloride toxicity suppresses osteogenic potential of rat bone marrow mesenchymal stem cells through reducing cell viability and bone matrix mineralization. Indian J Med Sci 65(4):157–167. 10.4103/0019-5359.104779 23250346

[CR51] Miyahara T, Takata M, Mori-Uchi S, Miyata M, Nagai M, Segure A, Matsusista M, Kozuka H, Kuze S (1992) Stimulative effects of cadmium on bone resorption in neonatal pariental bone resorption. Toxicology 73(1):93–99. 10.1016/0300-483X(92)90173-C 1589882 10.1016/0300-483x(92)90173-c

[CR52] Miyahara T, Yamada H, Takeuchi M, Kozuka H, Kato T, Sudo H (1988) Inhibitory effects of cadmium on in vitro calcification of a clonal osteogenic cell, MC3T3-E1. Toxicol Appl Pharmacol 96(1):52–59. 10.1016/0041-008X(88)90246-3 3188026 10.1016/0041-008x(88)90246-3

[CR53] Mobasheri A, Matta C, Uzielienè I, Budd E, Martín-Vasallo P, Bernotiene E (2019) The chondrocyte channelome: a narrative review. Joint Bone Spine 86(1):29–35. 10.1016/j.jbspin.2018.01.012 29452304 10.1016/j.jbspin.2018.01.012

[CR54] Moriguchi T, Yano K, Nakagawa S, Kaji F (2003) Elucidation of adsorption mechanism of bone-staining agent alizarin red S on hydroxyapatite by FT-IR microspectroscopy. J Colloid Interface Sci 260(1):19–25. 10.1016/S0021-9797(02)00157-1 12742030 10.1016/s0021-9797(02)00157-1

[CR55] Mork L, Crump G (2015) Zebrafish Craniofacial Development. A window into early patterning. In: Current Topics in Developmental Biology (1st ed vol 115). Elsevier Inc. 10.1016/bs.ctdb.2015.07.001 10.1016/bs.ctdb.2015.07.001 PMC4758817 26589928

[CR56] Movafeghi A, Khataee A, Abedi M, Tarrahi R, Dadpour M, Vafaei F (2018) Effects of TiO_2_ nanoparticles on the aquatic plant Spirodela polyrrhiza: evaluation of growth parameters, pigment contents and antioxidant enzyme activities. J Environ Sci (China) 64:130–138. 10.1016/j.jes.2016.12.020 29478632 10.1016/j.jes.2016.12.020

[CR57] Naasz S, Altenburger R, Kühnel D (2018) Environmental mixtures of nanomaterials and chemicals: the Trojan-horse phenomenon and its relevance for ecotoxicity. Sci Total Environ 635:1170–1181. 10.1016/j.scitotenv.2018.04.180 29710572 10.1016/j.scitotenv.2018.04.180

[CR58] OECD (2013) Test No. 236: fish embryo acute toxicity (FET) test. OECD Guidelines for the Testing of Chemicals, Section 2, OECD Publishing, July, 1–22. http://www.oecd-ilibrary.org. Accessed 3 Jul 2026

[CR59] Oger MJL, Bernay B, Tessier E, Amouroux D, Kestemont P, Cornet V (2025) The Trojan Horse effect of nanoplastics exacerbates methylmercury-induced neurotoxicity during zebrafish development. Environ Pollut 384:126966. 10.1016/j.envpol.2025.126966 40782993 10.1016/j.envpol.2025.126966

[CR60] Oliveira H, Monteiro C, Pinho F, Pinho S, Miguel J, De Oliveira PF (2014) Cadmium-induced genotoxicity in human osteoblast-like cells. Mutation Research / Genetic Toxicology and Environmental Mutagenesis 776:38–47. 10.1016/j.mrgentox.2014.10.002 10.1016/j.mrgentox.2014.10.002 25435354

[CR61] Pekey H, Karakaş D, Bakogˇlu M (2004) Source apportionment of trace metals in surface waters of a polluted stream using multivariate statistical analyses. Marine Pollution Bulletin 49(9–10):809–818. 10.1016/j.marpolbul.2004.06.029 15530525 10.1016/j.marpolbul.2004.06.029

[CR62] Pi H, Li M, Zou L, Yang M, Deng P, Fan T, Liu M, Tian L, Tu M, Xie J, Chen M, Li H, Xi Y, Zhang L, He M, Lu Y, Chen C, Zhang T, Wang Z, Yub Z, Gao F, Zhou Z (2019) AKT inhibition-mediated dephosphorylation of TFE3 promotes overactive autophagy independent of MTORC1 in cadmium-exposed bone mesenchymal stem cells. Autophagy 15(4):565–582. 10.1080/15548627.2018.1531198 10.1080/15548627.2018.1531198 PMC6526814 30324847

[CR63] Pinheiro-da-Silva J, Silva PF, Nogueira MB, Luchiari AC (2017) Sleep deprivation effects on object discrimination task in zebrafish (*Danio rerio*). Anim Cogn 20(2):159–169. 10.1007/s10071-016-1034-x 27646310 10.1007/s10071-016-1034-x

[CR64] Pinheiro-Da-Silva J, Agues-Barbosa T, Carolina Luchiari A (2020) Embryonic exposure to ethanol increases anxiety-like behavior in fry zebrafish. Alcohol Alcohol 55(6):581–590. 10.1093/ALCALC/AGAA087 32886092 10.1093/alcalc/agaa087

[CR65] Qian W, Chen CC, Zhou S, Huang Y, Zhu X, Wang Z, Cai Z (2020) TiO_2_ nanoparticles in the marine environment: enhancing bioconcentration, while limiting biotransformation of arsenic in the mussel *Perna viridis*. Environ Sci Technol 54(19):12254–12261. 10.1021/acs.est.0c01620 32866374 10.1021/acs.est.0c01620

[CR66] Rishi KK, Jain M (1998) Effect of toxicity of cadmium on scale morphology in *Cyprinus carpio* (Cyprinidae). Bull Environ Contam Toxicol 60(2):323–328. 10.1007/s001289900629 9470997 10.1007/s001289900629

[CR67] Rocco L, Santonastaso M, Nigro M, Mottola F, Costagliola D, Bernardeschi M, Guidi P, Lucchesi P, Scarcelli V, Corsi I, Stingo V, Frenzilli G (2015) Genomic and chromosomal damage in the marine mussel *Mytilus galloprovincialis*: effects of the combined exposure to titanium dioxide nanoparticles and cadmium chloride. Mar Environ Res 111:144–148. 10.1016/j.marenvres.2015.09.004 26392349 10.1016/j.marenvres.2015.09.004

[CR68] Rocha TL, Gomes T, Cardoso C, Letendre J, Pinheiro JP, Sousa VS, Teixeira MR, Bebianno MJ (2014) Immunocytotoxicity, cytogenotoxicity and genotoxicity of cadmiumbased quantum dots in the marine mussel *Mytilus galloprovincialis*. Mar Environ Res 101(1):29–37. 10.1016/j.marenvres.2014.07.009 25164019 10.1016/j.marenvres.2014.07.009

[CR69] Rodríguez J, Mandalunis PM (2016) Effect of cadmium on bone tissue in growing animals. Exp Toxicol Pathol 68(7):391–397. 10.1016/j.etp.2016.06.001 27312893 10.1016/j.etp.2016.06.001

[CR70] Ruvinda KMS, Pathiratne A (2021) Toxicity of titanium dioxide nanoparticles to tadpoles of Asian common toad (*Duttaphrynus melanostictus*) following short term and chronic exposures. Bull Environ Contam Toxicol 107:848–854. 10.1007/s00128-021-03352-y 34414477 10.1007/s00128-021-03352-y

[CR71] Sassi A, Annabi A, Kessabi K, Kerkeni A, Saïd K, Messaoudi I (2010) Influence of high temperature on cadmium-induced skeletal deformities in juvenile mosquitofish (*Gambusia affinis*). Fish Physiol Biochem 36(3):403–409. 10.1007/s10695-009-9307-9 19229646 10.1007/s10695-009-9307-9

[CR72] Sassi A, Darias MJ, Said K, Messaoudi I, Gisbert E (2013) Cadmium exposure affects the expression of genes involved in skeletogenesis and stress response in gilthead sea bream larvae. Fish Physiol Biochem 39(3):649–659. 10.1007/s10695-012-9727-9 23053610 10.1007/s10695-012-9727-9

[CR73] Schilling TF, Piotrowski T, Grandel H, Brand M, Heisenberg CP, Jiang YJ, Beuchle D, Hammerschmidt M, Kane DA, Mullins MC, Eeden FJ, Van M, Kelsh RN, Furutani-seiki M, Granato M, Haffter P, Odenthal J, Warga RM, Trowe T, Nüsslein-volhard C (1996) Jaw and branchial arch mutants in zebrafish I: branchial arches. Development 123:329–344. 10.1242/dev.123.1.329 10.1242/dev.123.1.329 9007253

[CR74] Scott JE, Dorling J (1965) Differential staining of acid glycosaminoglycans (mucopolysaccharides) by Alcian blue in salt solutions. Histochemie 5(3):221–233. 10.1007/BF00306130 4223499 10.1007/BF00306130

[CR75] Sloman KA, Scott GR, Diao Z, Rouleau C, Wood CM, McDonald DG (2003) Cadmium affects the social behaviour of rainbow trout, Oncorhynchus mykiss. Aquatic Toxicology 65(2):171–185. 10.1016/S0166-445X(03)00122-X 12946617 10.1016/s0166-445x(03)00122-x

[CR76] Slomberg DL, Auffan M, Guéniche N, Angeletti B, Campos A, Borschneck D, Aguerre-Chariol O, Rose J (2020) Anthropogenic release and distribution of titanium dioxide particles in a river downstream of a nanomaterial manufacturer industrial site. Front Environ Sci 8(June):1–12. 10.3389/fenvs.2020.00076

[CR77] Smith SS, Reyes JR, Arbon KS, Harvey WA, Hunt LM, Heggland SJ (2009) Cadmium-induced decrease in RUNX2 mRNA expression and recovery by the antioxidant N-acetylcysteine (NAC) in the human osteoblast-like cell line, Saos-2. Toxicol in Vitro 23(1):60–66. 10.1016/j.tiv.2008.10.011 19017541 10.1016/j.tiv.2008.10.011 PMC2644557

[CR78] Steward AJ, Kelly DJ, Wagner DR (2014) The role of calcium signalling in the chondrogenic response of mesenchymal stem cells to hydrostatic pressure. Eur Cells Mater 28(October):358–371. 10.22203/eCM.v028a25 10.22203/ecm.v028a25 25350251

[CR79] Strecker R, Weigt S, Braunbeck T (2013) Cartilage and bone malformations in the head of zebrafish (*Danio rerio*) embryos following exposure to disulfiram and acetic acid hydrazide. Toxicol Appl Pharmacol 268(2):221–231. 10.1016/j.taap.2013.01.023 23391615 10.1016/j.taap.2013.01.023

[CR80] Sukarman, Kristiawan B, Khoirudin, Abdulah A, Enoki K, Wijayanta AT (2024) Characterization of TiO2 nanoparticles for nanomaterial applications: crystallite size, microstrain and phase analysis using multiple techniques. Nano-Struct Nano-Objects 38:101168. 10.1016/j.nanoso.2024.101168

[CR81] Suthar S, Nema AK, Chabukdhara M, Gupta SK (2009) Assessment of metals in water and sediments of Hindon River, India: impact of industrial and urban discharges. Journal of Hazardous Materials 171(1–3):1088–1095. 10.1016/j.jhazmat.2009.06.109 19616893 10.1016/j.jhazmat.2009.06.109

[CR82] Suzuki N, Yamamoto M, Watanabe K, Kambegawa A, Hattori A (2004) Both mercury and cadmium directly influence calcium homeostasis resulting from the suppression of scale bone cells: the scale is a good model for the evaluation of heavy metals in bone metabolism. J Bone Miner Metab 22(5):439–446. 10.1007/s00774-004-0505-3 15316864 10.1007/s00774-004-0505-3

[CR83] Suzuki Y, Morita I, Yamane Y, Murota S (1990) Preventive effect of zinc against cadmium-induced bone resorption. Toxicology 62(1):27–34. 10.1016/0300-483X(90)90028-F 10.1016/0300-483x(90)90028-f 2343456

[CR84] Tarasco M, Cardeira J, Viegas MN, Caria J, Martins G, Gavaia PJ, Cancela ML, Laizé V (2019) Anti-osteogenic activity of cadmium in Zebrafish. Fishes 4(1):1–15. 10.3390/fishes4010011

[CR85] Tian J, Hu J, He W, Zhou L, Huang Y (2020) Parental exposure to cadmium chloride causes developmental toxicity and thyroid endocrine disruption in zebrafish offspring. Comparative Biochemistry and Physiology Part - C: Toxicology and Pharmacology 234(April):108782. 10.1016/j.cbpc.2020.108782 32339758 10.1016/j.cbpc.2020.108782

[CR86] Tu H, Fan C, Chen X, Liu J, Wang B, Huang Z, Zhang Y, Meng X, Zou F (2017) Effects of cadmium, manganese, and lead on locomotor activity and neurexin 2a expression in zebrafish. Environ Toxicol Chem 36(8):2147–2154. 10.1002/etc.3748 28120348 10.1002/etc.3748

[CR87] Uzieliene I, Bernotas P, Mobasheri A, Bernotiene E (2018) The role of physical stimuli on calcium channels in chondrogenic differentiation of mesenchymal stem cells. Int J Mol Sci 19(10). 10.3390/ijms19102998 10.3390/ijms19102998 PMC6212952 30275359

[CR88] Waghmode MS, Gunjal AB, Mulla JA, Patil NN, Nawani NN (2019) Studies on the titanium dioxide nanoparticles: biosynthesis, applications and remediation. SN Applied Sciences 1(4):310. 10.1007/s42452-019-0337-3

[CR89] Wagner EF, Karsenty G (2001) Genetic control of skeletal development. Curr Opin Genet Dev 11(5):527–532. 10.1016/S0959-437X(00)00228-8 11532394 10.1016/s0959-437x(00)00228-8

[CR90] Walker MB, Kimmel CB (2007) A two-color acid-free cartilage and bone stain for zebrafish larvae. Biotech Histochem 82(1):23–28. 10.1080/10520290701333558 17510811 10.1080/10520290701333558

[CR91] Wilson AK, Cerny EA, Smith BD, Wagh A, Bhattacharyya MH (1996) Effects of cadmium on osteoclast formation and activity in vitro. Toxicol Appl Pharmacol 140(2):451–460. 10.1006/taap.1996.0242 8887463 10.1006/taap.1996.0242

[CR92] Wong CKC, Wong MH (2000) Morphological and biochemical changes in the gills of Tilapia (*Oreochromis mossambicus*) to ambient cadmium exposure. Aquat Toxicol 48(4):517–527. 10.1016/S0166-445X(99)00060-0 10794834 10.1016/s0166-445x(99)00060-0

[CR93] Wright DA, Welbourn PM (1994) Cadmium in the aquatic environment: a review of ecological, physiological, and toxicological effects on biota. Environ Rev 2(2):187–214. 10.1139/a94-012

[CR94] Wu SM, Su CK, Shu LH (2018) Effects of calcium and estrogen on the development of the ceratohyal cartilage in zebrafish (*Danio rerio*) larvae upon embryo and maternal cadmium exposure. Comparative Biochemistry and Physiology Part - C: Toxicology and Pharmacology 213(May):47–54. 10.1016/j.cbpc.2018.07.006 30059766 10.1016/j.cbpc.2018.07.006

[CR95] Wu L, Wei Q, Lv Y, Xue J, Zhang B, Sun Q, Xiao T, Huang R, Wang P, Dai X, Xia H, Li J, Yang X, Liu Q (2019) Wnt/β-catenin pathway is involved in cadmium-induced inhibition of osteoblast differentiation of bone marrow mesenchymal stem cells. Int J Mol Sci 20(6). 10.3390/ijms20061519 10.3390/ijms20061519 PMC6471709 30917596

[CR96] Xu F (2018) Review of analytical studies on TiO_2_ nanoparticles and particles aggregation, coagulation, flocculation, sedimentation, stabilization. Chemosphere 212:662–677. 10.1016/j.chemosphere.2018.08.108 30173113 10.1016/j.chemosphere.2018.08.108

[CR97] Yang M, Pi H, Li M, Xu S, Zhang L, Xie J, Tian L, Tu M, He M, Lu Y, Yu Z, Zhou Z (2016) Autophagy induction contributes to cadmium toxicity in mesenchymal stem cells via AMPK/FOXO3a/BECN1 signaling. Toxicol Sci 154(1):101–114. 10.1093/toxsci/kfw144 27492225 10.1093/toxsci/kfw144

[CR98] Yao H, Qian X, Gao H, Wang Y, Xia B (2014) Seasonal and Spatial Variations of heavy metals in two typical Chinese rivers: concentrations, environmental risks, and possible sources. International Journal of Environmental Research and Public Health 11(11):11860–11878. 10.3390/ijerph111111860 25407421 10.3390/ijerph111111860 PMC4245648

[CR99] Yoshitomi T, Koyama J, Iida A, Okamoto N, Ikeda Y (1998) Cadmium-induced scale deformation in Carp (*Cyprinus carpio*). Bull Environ Contam Toxicol 60(4):639–644. 10.1007/s001289900674 9557205 10.1007/s001289900674

[CR100] Zhang X, Sun H, Zhang Z, Niu Q, Chen Y, Crittenden JC (2007) Enhanced bioaccumulation of cadmium in carp in the presence of titanium dioxide nanoparticles. Chemosphere 67(1):160–166. 10.1016/j.chemosphere.2006.09.003 17166554 10.1016/j.chemosphere.2006.09.003

[CR101] Zhao H, Liu W, Wang Y, Dai N, Gu J, Yuan Y, Liu X, Bian J, Liu ZP (2015) Cadmium induces apoptosis in primary rat osteoblasts through caspase and mitogen-activated protein kinase pathways. J Vet Sci 16(3):297–306. 10.4142/jvs.2015.16.3.297 26425111 10.4142/jvs.2015.16.3.297 PMC4588015

[CR102] Zheng JL, Yuan SS, Wu CW, Lv ZM (2016) Acute exposure to waterborne cadmium induced oxidative stress and immunotoxicity in the brain, ovary and liver of zebrafish (*Danio rerio*). Aquat Toxicol 180:36–44. 10.1016/j.aquatox.2016.09.012 27642707 10.1016/j.aquatox.2016.09.012

[CR103] Zhou Z, Liu T, Kong J, Zeng S, Zhu J (2024) Toxicity and transcriptome sequencing analyses of *Acipenser schrenckii* under titanium dioxide nanoparticles stress. Aquaculture Reports 36(818):102091. 10.1016/j.aqrep.2024.102091

[CR104] Zhu X, Chang Y, Chen Y (2010) Toxicity and bioaccumulation of TiO_2_ nanoparticle aggregates in Daphnia magna. Chemosphere 78(3):209–215. 10.1016/j.chemosphere.2009.11.013 19963236 10.1016/j.chemosphere.2009.11.013

